# Eosinophils in autoimmune diseases: context-dependent pathogenesis, functional heterogeneity, and therapeutic translation

**DOI:** 10.3389/fimmu.2026.1962099

**Published:** 2026-10-19

**Authors:** Zhidan Fan, Wenjing Zhu, Na Huang, Haiguo Yu

**Affiliations:** Department of Rheumatology and Immunology, Children's Hospital of Nanjing Medical University, Nanjing, China

**Keywords:** autoimmune diseases, biologics, cellular heterogeneity, EETosis, eosinophils, IL-33/ST2, type 2 inflammation

## Abstract

Eosinophils are best known for their host defence against helminths and for their effector role in type 2 inflammation, but an expanding evidence base indicates that their functions extend to immune regulation, tissue repair, fibrosis, and selected autoimmune phenotypes. This narrative review critically evaluates eosinophil biology across inflammatory arthritis, systemic lupus erythematosus (SLE), antineutrophil cytoplasmic antibody-associated vasculitis (AAV), autoimmune neurological and endocrine disorders, autoimmune dermatoses, inflammatory bowel disease (IBD), and fibrosing connective-tissue disorders. We organize the evidence around four recurring processes: tissue recruitment and niche imprinting, degranulation and eosinophil extracellular trap formation, cross talk with innate and adaptive immune cells, and context-dependent repair or fibrotic remodelling. The strength of evidence is highly disease dependent. Human mechanistic and interventional data are strongest in eosinophilic granulomatosis with polyangiitis (EGPA) and selected eosinophil-rich dermatoses, whereas many proposed roles in RA, SLE, central nervous system autoimmunity, endocrinopathies, and IBD remain observational or preclinical. Proposed resident/regulatory and inflammatory eosinophil states support functional plasticity, but several frequently used marker schemes derive predominantly from mouse studies and do not yet constitute a standardized human taxonomy. Therapeutically, IL-5/IL-5Rα blockade provides clinically validated benefit in EGPA, but efficacy should not be extrapolated across autoimmune diseases without phenotype-specific evidence. Transient blood eosinophilia and rare eosinophilic complications during IL-4Rα blockade are clinically recognized; however, compensatory IL-5 signaling, altered trafficking, and unmasking of pre-existing eosinophilic disease remain competing, incompletely resolved explanations. Across the field, causal inference is constrained by species differences, limited access to representative human tissue, heterogeneous biomarker definitions, and a scarcity of controlled trials. Eosinophils should therefore be viewed not as universal drivers of autoimmunity but as context-dependent immune modulators whose pathogenic or protective contribution varies by tissue, disease stage, and cellular state. Human tissue–resolved studies and phenotype-guided trials will be essential for translating this heterogeneity into precision therapy.

## 1 Introduction

Autoimmune diseases arise from failures of self-tolerance combined with tissue-specific inflammatory programs and encompass disorders as diverse as rheumatoid arthritis (RA), SLE, vasculitis, autoimmune neurological disease, inflammatory bowel disease, and autoimmune dermatoses (1–6). Although adaptive autoreactivity remains central to many of these conditions, tissue-resident and -recruited innate immune cells can shape the threshold, localization, persistence, and resolution of inflammation. Eosinophils (EOS) are particularly relevant to this interface because their effector repertoire includes preformed granule proteins, lipid mediators, cytokines, extracellular traps, and signals that influence epithelium, endothelium, stromal cells, T cells, B cells, and myeloid populations (7–16).

Recent single-cell, spatial, genetic, and interventional studies have challenged the assumption that EOS are a homogeneous terminal effector population. Tissue niches can imprint distinct activation states, and EOS may contribute to barrier defence and repair in one context while promoting cytotoxicity, thrombosis, fibrosis, or inflammatory amplification in another (11–15, 17–19). This functional plasticity is important for autoimmune disease, but the evidence is uneven: some conclusions are supported by human tissue or controlled trials, whereas others rely on mouse models, *in-vitro* systems, or small observational cohorts. Accordingly, throughout this review, we distinguish association from causation and label the evidentiary basis of major mechanistic claims.

We use the terms autoimmune and autoinflammatory non-interchangeably. Autoimmune disease is defined here by adaptive autoreactivity, including autoreactive lymphocytes and/or autoantibodies, whereas autoinflammatory disease is dominated by dysregulated innate sensing and cytokine pathways. EOS are not canonical inflammasome-driven cells; nevertheless, epithelial alarmins and IL-1-family signals, especially IL-33, can connect sterile tissue injury to type 2 and eosinophilic programs. This overlap is discussed where it informs mechanism, without reclassifying classical autoimmune diseases as autoinflammatory disorders.

The central question of this review is therefore not whether EOS are globally “pathogenic” or “protective,” but under which tissue, disease-stage, and cellular-state conditions each function emerges. We compare eight disease clusters using a common framework: recruitment and tissue imprinting; effector injury through degranulation, reactive oxygen species (ROS), and eosinophil extracellular traps (EETs); innate–adaptive immune cross talk; and repair/fibrotic remodelling. We then evaluate biomarker and therapeutic implications against the strength and limitations of the supporting evidence.

Review approach and literature selection. This article is a structured narrative review rather than a systematic review or meta-analysis. Literature identification used iterative PubMed/MEDLINE searches supplemented by citation chaining and targeted searches for disease-specific mechanisms and therapeutics. Search concepts combined “eosinophil*” with terms including “autoimmun*,” individual disease names, “EETosis/EETs,” “IL-33/ST2,” “IL-5/IL-5R,” “Siglec-8,” “extracellular vesicle*,” “single-cell,” “spatial,” and “Mendelian randomization.” The bibliography was updated through September 2026. Priority was given to primary human studies, controlled trials, meta-analyses, high-quality mechanistic studies, and recent single-cell or spatial studies; duplicate, non-relevant, and purely descriptive reports without mechanistic or clinical relevance were not prioritized. Because study designs and outcomes differ substantially across diseases, we did not calculate *de-novo* pooled effect estimates; instead, representative quantitative human findings are summarized in **Table 1**.

**Table 1:** Selected quantitative human evidence anchoring major clinical claims.

Disease	Study design	Population	Quantitative finding	Interpretation/evidence boundary
RA	Two-sample Mendelian randomization	14,361 cases; 43,923 controls; 368 eosinophil-count instruments	IVW OR 1.58 (95% CI 1.39–2.68); validation OR 1.21 (1.04–1.40)	Genetic-instrument association; not a cell-level mechanism (20)
SLE activity	Cross-sectional ROC study	83 SLE (40 flare, 43 remission) + 85 controls	ELR ≤ 0.0076: flare vs. remission AUC 0.647; ELR ≤ 0.029: flare vs. controls AUC 0.775	Cohort-specific thresholds; not validated for lupus nephritis (21)
Psoriasis	Systematic review/meta-analysis of diagnostic pathology studies	5 studies; pooled data 218 cases	Dermal EOS pooled prevalence 46% (95% CI 27%–66%); *I*² = 89%	High heterogeneity; prevalence does not establish severity correlation (22)
EGPA	Randomized placebo-controlled MIRRA trial	136 patients (68/arm)	≥ 24 weeks accrued remission: 28% vs. 3%; OR 5.91 (2.68–13.03)	Controlled clinical evidence for mepolizumab in relapsing/refractory EGPA (23)
EGPA	Randomized active-comparator MANDARA trial	140 patients	Remission at weeks 36 & 48: ~59% benralizumab vs. 56% mepolizumab; noninferior	Controlled clinical evidence; does not prove one eosinophil subset mediates response (24)

Inhaled allergen exposure initiates epithelial-derived alarmins-dominated type-2 inflammatory cascades in upper airway mucosa (**Figure 1**).

**Figure 1:**
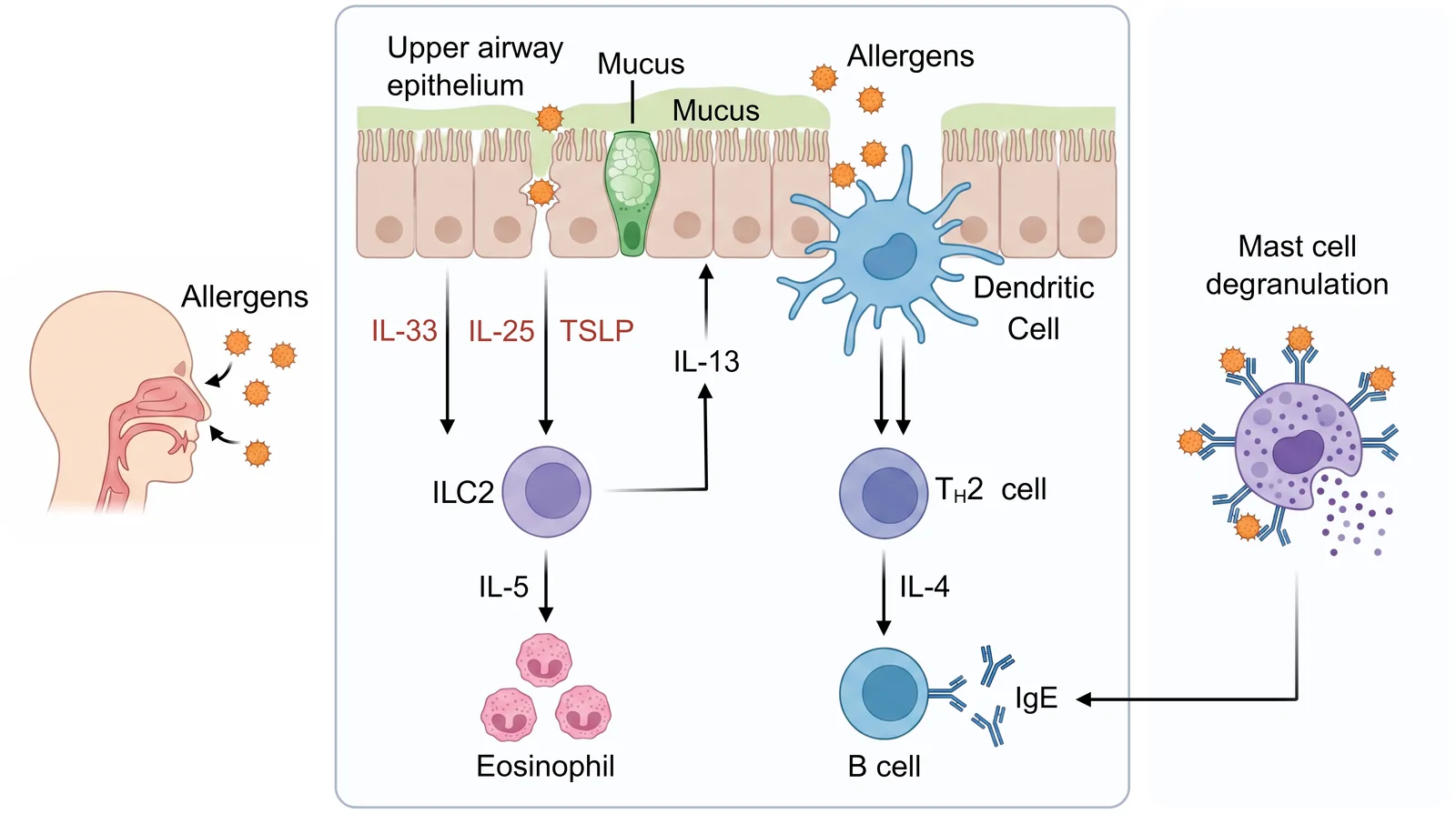
Type 2 immune circuitry initiated by airborne allergens at the upper-airway barrier. Created in BioRender. Zhu, W. (2026) https://BioRender.com/i3l3p9o.

Epithelial alarmins (TSLP, IL-25, and IL-33) activate ILC2s, while antigen presentation promotes T-helper-2 (Th2) differentiation. ILC2- and Th2-derived cytokines support eosinophil recruitment, IgE production, mast-cell activation, mucus production, and barrier inflammation. ILC2 denotes the innate component of type 2 immunity, whereas Th2 denotes the adaptive CD4+ T-cell component. Created with .

## 2 Biological characteristics, functional heterogeneity, and immunoregulatory overview of eosinophils

EOS are a granulocyte subset originating from bone marrow myeloid progenitors, possessing a unique granular structure and multifunctional immune activity. Since their first discovery by pathologists in the 19th century, the cognitive paradigm has undergone multiple subversive iterations: from initially being considered purely as parasite-defending effector cells and hallmark cells of type 2 inflammation, they have progressively been redefined as multifunctional core immune cells with roles in homeostatic regulation, immune modulation, tissue remodelling, and the driving of autoimmune pathology, emerging as a key hub cell connecting innate immunity, adaptive immunity, and tissue microenvironment homeostasis imbalance (25, 26). Traditional immunological systems long regarded EOS as terminally differentiated, functionally singular, passive effector cells. However, over the past decade, single-cell transcriptomics, spatial immunohistochemistry, gene knockout animal models, and targeted depletion clinical studies have confirmed that EOS possess significant tissue-specific plasticity, subset heterogeneity, and functional bidirectionality. They play markedly different, even entirely antagonistic, biological roles in physiological homeostasis versus autoimmune, chronic inflammatory, and fibrotic diseases, fundamentally reshaping the traditional perception of these cells (27–29). This section systematically elaborates on the foundational biological panorama of EOS from multiple dimensions—including the evolution of their discovery, developmental and differentiation regulation, morphological and structural characteristics, tissue distribution and homeostatic residency, subset heterogeneity, activation modes, core biological functions, and cognitive controversies and research limitations—laying the theoretical groundwork for the subsequent dissection of their pathogenesis in autoimmune diseases.

### 2.1 Evolution of the eosinophil concept

Paul Ehrlich named EOS in 1878 on the basis of their eosinophilic granules (30, 31). For much of the 20th century, they were viewed primarily as terminal effectors in helminth defence and allergic inflammation (32, 33). Molecular immunology, targeted depletion studies, and more recently single-cell and spatial approaches have broadened this model by showing that EOS can participate in innate defence, tissue remodelling, immune regulation, and context-dependent inflammatory injury (26, 27, 34–39). The modern view is therefore one of tissue-adapted functional heterogeneity rather than a fixed pro-inflammatory phenotype; however, many proposed subsets remain incompletely validated in humans.

### 2.2 Developmental differentiation and survival regulation mechanisms of eosinophils

EOS arise from CD34+ haematopoietic progenitors and mature through eosinophil-committed intermediates under coordinated transcriptional and cytokine control. IL-5 is particularly important for terminal maturation, survival, and mobilization, while PU.1, GATA-family factors, and C/EBPϵ contribute to lineage specification and granule-gene programs (40–43). These pathways provide the biological rationale for IL-5/IL-5R–directed therapies, but IL-5 dependence varies by tissue state and does not imply that all eosinophil functions are therapeutically interchangeable.

After bone marrow egress, eosinophil trafficking is shaped by CCR3 ligands (including CCL11, CCL24, and CCL26), adhesion molecules, epithelial alarmins, and tissue-derived survival signals. Local stromal, epithelial, neural, microbial, and metabolic cues can alter activation thresholds and mediator release, linking development to tissue-specific function (40–47). Eosinophil development, egress from bone marrow and release of granule-associated effector molecules underpin their multifaceted immune functions (**Figure 2**).

**Figure 2:**
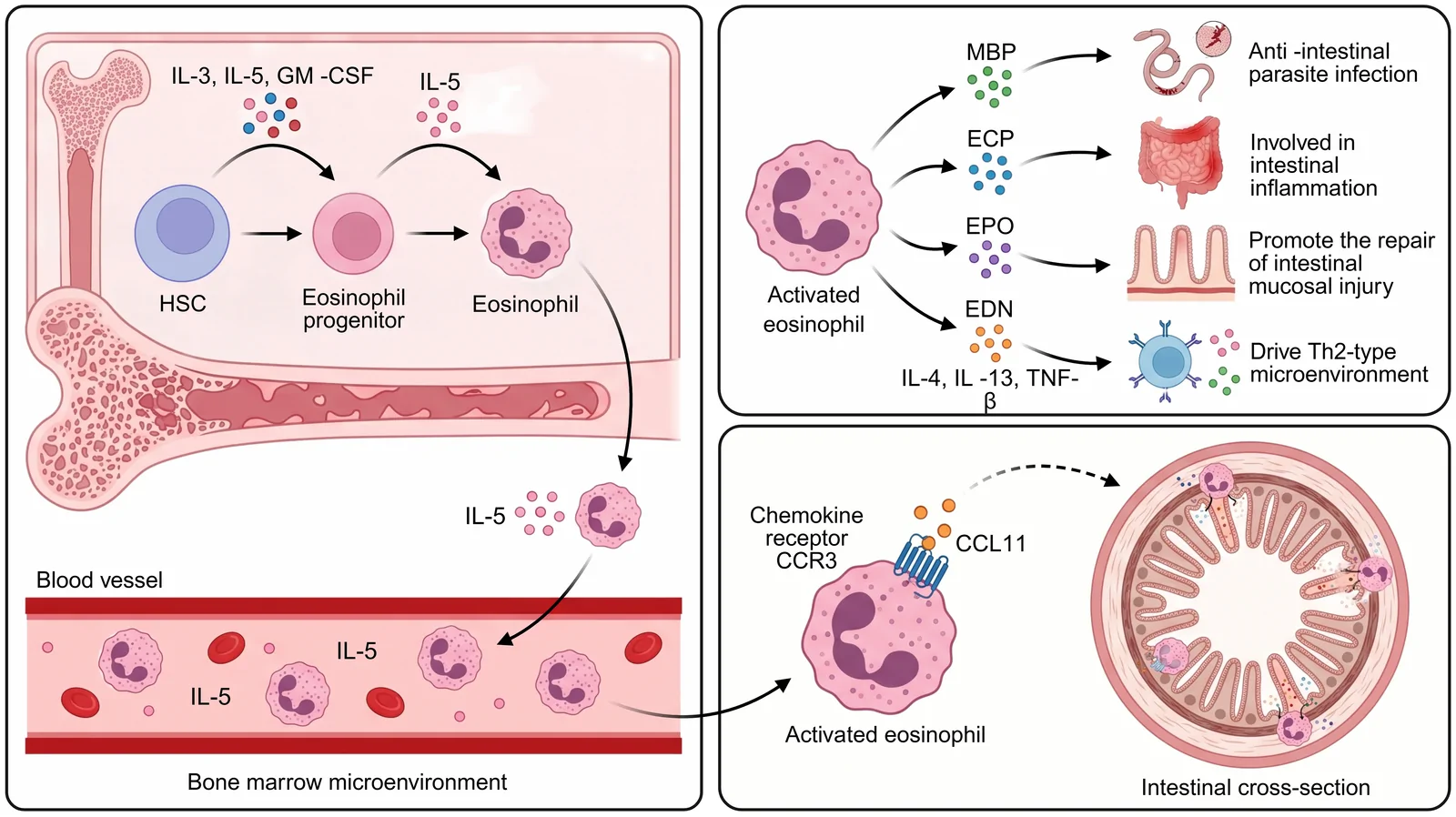
Eosinophil development, trafficking, and representative effector outputs. Created in BioRender. Zhu, W. (2026) https://BioRender.com/1vjyh68.

IL-3, IL-5, and GM-CSF support eosinophil development; IL-5 is especially important for terminal maturation and survival. CCR3–eotaxin signaling contributes to tissue recruitment. Activated EOS can release MBP, ECP, EPO, EDN, TNF-α, and type 2 cytokines, with context-dependent effects on host defence, inflammation, and tissue repair. Created with .

After egressing from the bone marrow, mature EOS retain only a small fraction in the peripheral blood circulation (normally accounting for 1%–6% of total white blood cells in humans). The vast majority migrate to mucosal tissues throughout the body via specific chemotactic signals, with the gastrointestinal tract, respiratory tract, skin, and perivascular tissues as their primary sites of residence. Notably, the gastrointestinal mucosa, excluding the oesophagus, is the largest site of homeostatic eosinophil residency in the human body (44, 45). Under physiological conditions, tissue-resident EOS are characterized by a long lifespan, low proliferation, and a quiescent state, maintaining tissue microenvironment homeostasis through autocrine and paracrine signals. Under pathological conditions, chemokines such as IL-5, eotaxin (CCL11), CCL24, and CCL26 massively recruit peripheral blood EOS to inflammatory sites while simultaneously prolonging the lifespan of local EOS, disrupting the homeostatic balance and triggering massive local cell infiltration and activation (46). Diverse niche-derived cues from stroma, epithelium, microbiota, and nerves collectively shape eosinophil functional states (**Figure 3**).

**Figure 3:**
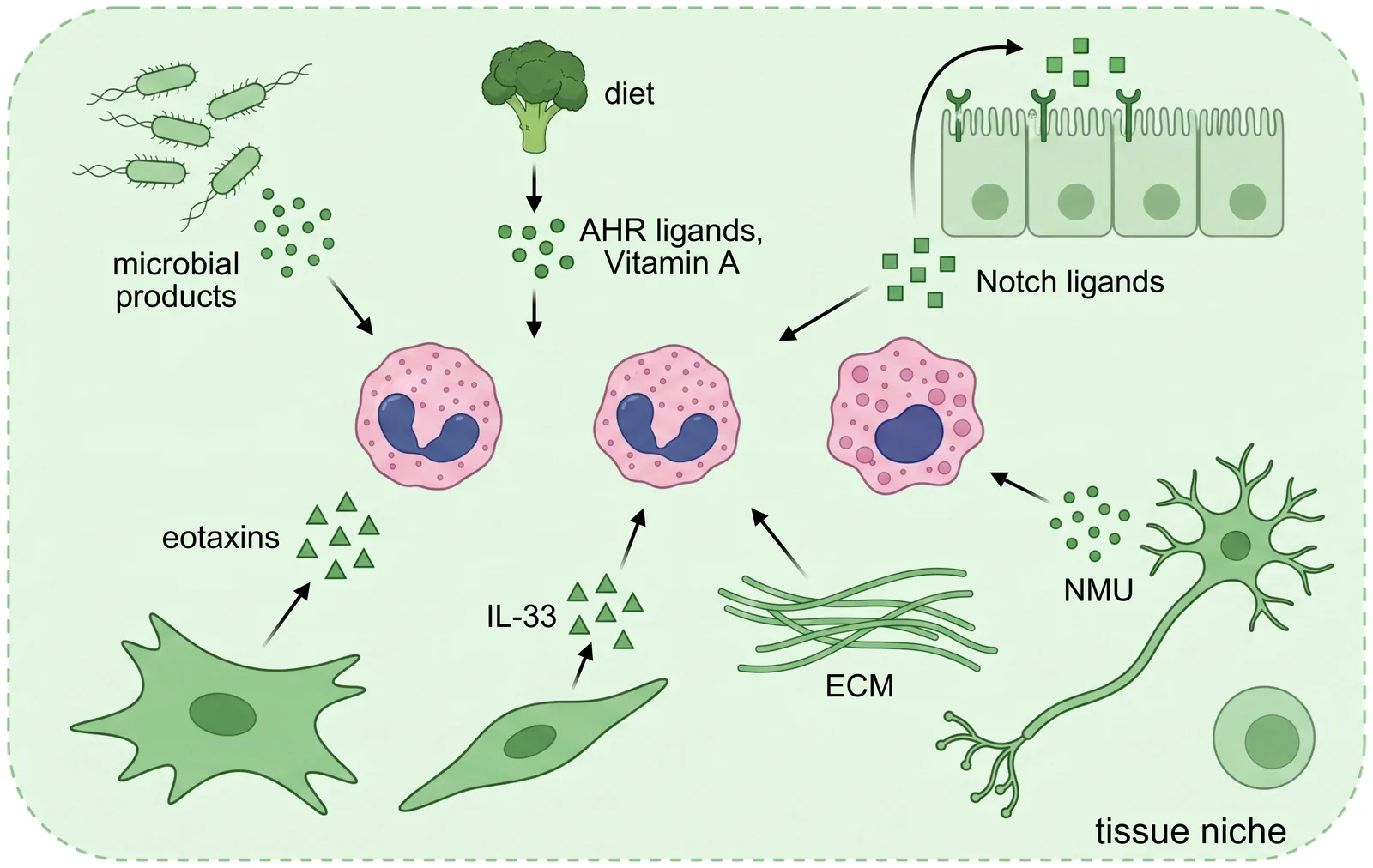
Tissue-niche signals that shape eosinophil plasticity. Created in BioRender. Zhu, W. (2026) https://BioRender.com/0c5kowm.

Microbial metabolites, dietary cues, stromal eotaxins and IL-33, extracellular–matrix signals, epithelial ligands, and neuroimmune mediators can modify eosinophil state and function. These inputs should be interpreted as context-setting signals rather than a single conserved differentiation program. Created with .

Eosinophil survival and activation are highly context dependent. Cytokines and danger signals can prolong survival and alter degranulation, oxidative responses, and transcriptional programs, whereas epigenetic regulation may contribute to longer lived state changes (40, 47). Purinergic signaling provides an additional extracellular signaling mechanism regulating eosinophil activation and mediator release (48). Evidence for durable “trained” eosinophil immunity remains emerging; the 2023 study by Gurtner et al. instead provides strong evidence for tissue-adapted active eosinophil states in intestinal inflammation and host defence (49). Thus, memory-like terminology should be used cautiously until durable, stimulus-specific reprogramming is demonstrated in human EOS.

### 2.3 Morphology, granule biology, extracellular traps, and vesicular communication

EOS possess a unique morphology and intracellular structure distinct from other granulocytes. Their most defining feature is the abundance of specific eosinophilic granules within the cytoplasm, which serve as the core vehicle for their biological functions. Mature human EOS are round granulocytes with a diameter of 10–12 μm, typically featuring a bilobed nucleus with dense chromatin. The cytoplasm is filled with coarse, uniform, orange–red specific granules that stain intensely with eosin, along with a small number of primary granules and vesicular structures (31). Compared with mouse EOS, human EOS specifically express Charcot–Leyden crystal (CLC) protein (galectin-10), a hallmark characteristic of human EOS and an important mediator and biomarker of inflammatory injury (50, 51).

The intracellular granules of EOS are divided into specific secondary granules and primary granules, storing dozens of biologically active toxic proteins, cytokines, chemokines, and lipid mediators. This constitutes the material basis for their multifunctional effects, and different activation modes can mediate the selective release of different mediators, enabling precise functional regulation (52). Specific secondary granules are the most abundant and functionally central granules, primarily containing four classical toxic proteins: eosinophil cationic protein (ECP), eosinophil-derived neurotoxin (EDN), eosinophil peroxidase (EPO), and major basic protein (MBP) (37). Among these, MBP is the most specific marker of EOS, possessing extremely strong cytotoxicity. It can directly damage the cell membranes of epithelial cells, endothelial cells, and target cells, inducing apoptosis and tissue injury. ECP and EDN possess both cytotoxic and immunomodulatory functions, regulating the activation of T cells and dendritic cells and participating in immune response regulation. EPO can catalyze the generation of reactive oxygen species, amplifying oxidative stress damage and mediating chronic inflammation and tissue fibrosis (53).

In addition to classical granule proteins, eosinophil granules and intracellular vesicles also store a large number of pro-inflammatory and immunoregulatory mediators, including type 2–associated cytokines (IL-4, IL-5, and IL-13), pro-inflammatory factors (TNF-α, IL-1β, and IL-6), anti-inflammatory factors (IL-10 and TGF-β), chemokines (CCL5 and CXCL8), and lipid mediators (leukotriene C4, prostaglandins, and platelet-activating factor) (54, 55). This characteristic of simultaneously storing pro-inflammatory and anti-inflammatory mediators is the core material basis for the bidirectional functionality of EOS. Their ultimate functional phenotype is entirely determined by the regulation of microenvironmental signals, explaining their drastically different roles in homeostasis versus disease (56). Recent airway-focused literature also highlights eicosanoid metabolism as an important inflammatory signaling layer relevant to eosinophil biology, although these data should not be extrapolated directly to autoimmune disease (57).

Eosinophil extracellular trap cell death (EETosis), discovered in recent years, is a unique activation-associated death mode of EOS, distinct from traditional cell death modalities like apoptosis and necrosis (58). Activated EOS can release extracellular traps (EETs) composed of DNA, histones, and granular toxic proteins. On one hand, EETs can capture and clear pathogens, participating in innate immune defence; on the other hand, they can release large amounts of CLCs and free granule proteins, persistently stimulating local inflammatory responses, inducing epithelial damage, fibrosis, and self-antigen exposure, thereby breaking immune tolerance. This makes EETosis a key mechanism in the initiation and sustained progression of autoimmune diseases (51, 58). Clinical studies have confirmed that the level of EETosis activation is closely correlated with the severity and prognosis of various autoimmune and eosinophilic diseases, serving as a novel disease biomarker (59).

Beyond soluble mediators and extracellular traps, EOS release extracellular vesicles (EVs) containing proteins, lipids, messenger RNAs, and microRNAs. Human eosinophil-derived EVs can participate in autocrine signaling and modify recipient structural or immune cells, with the best developed evidence coming from asthma and other eosinophilic disorders (60). Their relevance to classical autoimmune disease is plausible but remains largely untested in disease-specific human tissue. EVs should therefore be considered an emerging communication pathway and biomarker/therapeutic candidate rather than an established autoimmune mechanism.

### 2.4 Tissue distribution, homeostatic functions, and physiological regulatory roles of eosinophils

Under physiological homeostasis, EOS are cells for mucosal immunity and tissue homeostasis regulation. Their tissue distribution is highly specific and regular, barely participating in peripheral blood circulatory immune responses and primarily residing in mucosal barrier tissues, where they participate in barrier maintenance, microbial regulation, tissue repair, and immune tolerance maintenance (35, 44). Normal human peripheral blood eosinophil counts are maintained at (0.02–0.52) × 10^9^/L, an extremely low number in peripheral blood, whereas the number of tissue-resident EOS is dozens of times higher, and the phenotype and function of tissue-resident EOS in each organ exhibit significant tissue specificity (61).

The gastrointestinal tract is the primary homeostatic residence organ for EOS. Except for the esophageal mucosa, which lacks eosinophil infiltration under homeostasis, the lamina propria of the stomach, small intestine, and colon all contain stable eosinophil populations, displaying a numerical gradient distribution along the intestinal axis (45, 62). Homeostatic gut EOS do not trigger inflammatory responses but instead maintain intestinal microenvironment homeostasis through three functions: first, regulating intestinal barrier integrity by secreting low concentrations of TGF-β and IL-10 to maintain epithelial cell tight junctions and reduce intestinal permeability damage; second, regulating gut microbiota homeostasis by inhibiting excessive proliferation of harmful bacteria through antimicrobial mediators, maintaining microbial balance; and third, participating in intestinal tissue repair and metabolic regulation, promoting mucosal damage repair, modulating local intestinal immune tolerance, and preventing the initiation of aberrant inflammatory responses (46, 63). The homeostatic imbalance and aberrant activation of intestinal EOS are closely related to autoimmune-associated intestinal diseases such as inflammatory bowel disease and eosinophilic gastroenteritis (64, 65). A panel of core transcription factors endows intestinal EOS with tissue-specific transcriptomic and functional imprints (**Figure 4**).

**Figure 4:**
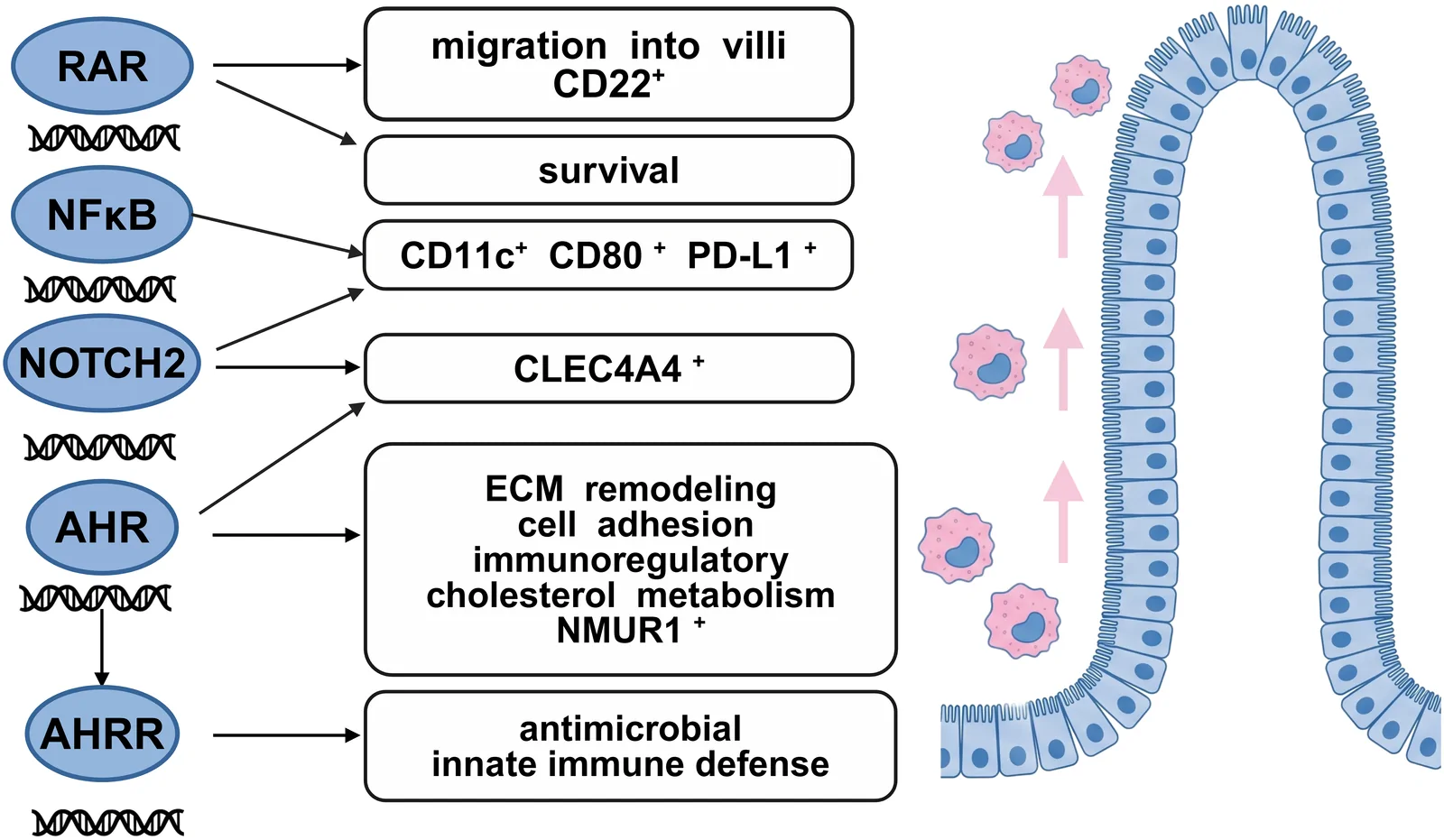
Transcriptional regulators associated with tissue adaptation of intestinal eosinophils. Created in BioRender. Zhu, W. (2026) https://BioRender.com/egay5bl.

RAR, NF-κB, NOTCH2, AHR, and AHRR-associated programs have been linked to intestinal eosinophil recruitment, survival, immune regulation, metabolism, and tissue adaptation. These signatures are context dependent and should not be interpreted as a universal transcriptional taxonomy. Created with .

The respiratory tract, skin, and urogenital system mucosae also harbor homeostatic EOS, participating in mucosal barrier defence and immune homeostasis regulation (66). Lung tissue–resident EOS can clear trace airway pathogens, regulate airway epithelial repair, and suppress excessive inflammatory responses. Skin EOS are involved in maintaining the skin barrier, wound healing, and local immune tolerance regulation, preventing the occurrence of cutaneous autoimmune reactions (53, 67). Furthermore, recent studies have discovered that EOS can reside in non-mucosal tissues such as adipose tissue, blood vessels, and bone, participating in metabolic regulation, vascular homeostasis maintenance, and bone metabolism modulation, expanding the cognitive boundaries of their physiological functions (68).

From a holistic physiological function perspective, the role of homeostatic EOS is immune fine-tuning and tissue homeostasis maintenance, rather than inflammatory injury (26). They suppress excessive immune responses by secreting anti-inflammatory factors, maintain peripheral immune tolerance by modulating the function of antigen-presenting cells, and maintain organ structural integrity by participating in tissue repair. Previous research has excessively focused on their pathological injury functions, neglecting their physiological protective roles. In recent years, clinical studies involving eosinophil depletion have found that long-term eosinophil deficiency can lead to impaired barrier function, microbial dysbiosis, and imbalanced immune tolerance, increasing the risk of infections and autoimmune diseases, directly confirming their indispensable physiological homeostatic functions (69, 70).

### 2.5 Eosinophil heterogeneity: proposed subsets, markers, and evidentiary boundaries

Eosinophil heterogeneity is supported by transcriptional, phenotypic, and functional studies, but there is no universally accepted human rEOS/iEOS taxonomy (71). Frequently cited CD62L/CD101 schemes derive largely from mouse lung studies, and markers such as CD11c, PD-L1, and NMUR1 can be tissue- or activation-context dependent rather than lineage-defining. **Table 1** therefore lists candidate phenotypes together with species and evidence-level caveats; these markers should not be treated as interchangeable diagnostic labels in human autoimmune disease. A recent review synthesizing eosinophil diversity and therapeutic targeting further emphasizes the need to distinguish subset concepts from validated human markers (72).

The currently recognized core classification system divides EOS into two major subsets based on activation status and function: tissue-resident eosinophils (rEOS) and inflammatory activated eosinophils (iEOS). These two subsets dynamically transition between physiological homeostasis and pathological states, with completely antagonistic functions (39, 73). rEOS are the predominant subset under physiological homeostasis, exhibiting low expression of pro-inflammatory mediators and high expression of anti-inflammatory and repair factors. Their functions are maintaining tissue homeostasis, promoting damage repair, and sustaining immune tolerance. They have low proliferative activity, a long lifespan, and no tissue-damaging capacity, primarily residing in homeostatic tissues such as the gastrointestinal tract, normal airways, and skin mucosa (67, 74). iEOS are an activated subset induced by pathological stimulation, derived from peripheral blood recruitment and local proliferation and transformation. They highly express pro-inflammatory cytokines, granular toxic proteins, and chemokines, possessing extremely strong pro-inflammatory, pro-fibrotic, and tissue-damaging capabilities, making them the core pathogenic subset in various eosinophilic inflammatory and autoimmune diseases (39). In diseases such as chronic rhinosinusitis, asthma, and intestinal inflammation, the phenotypic switch from rEOS to iEOS is a key molecular event in disease initiation and progression (75). Distinct eosinophil subsets occupy divergent anatomical sites under homeostatic and inflammatory disease conditions (**Figure 5**).

**Figure 5:**
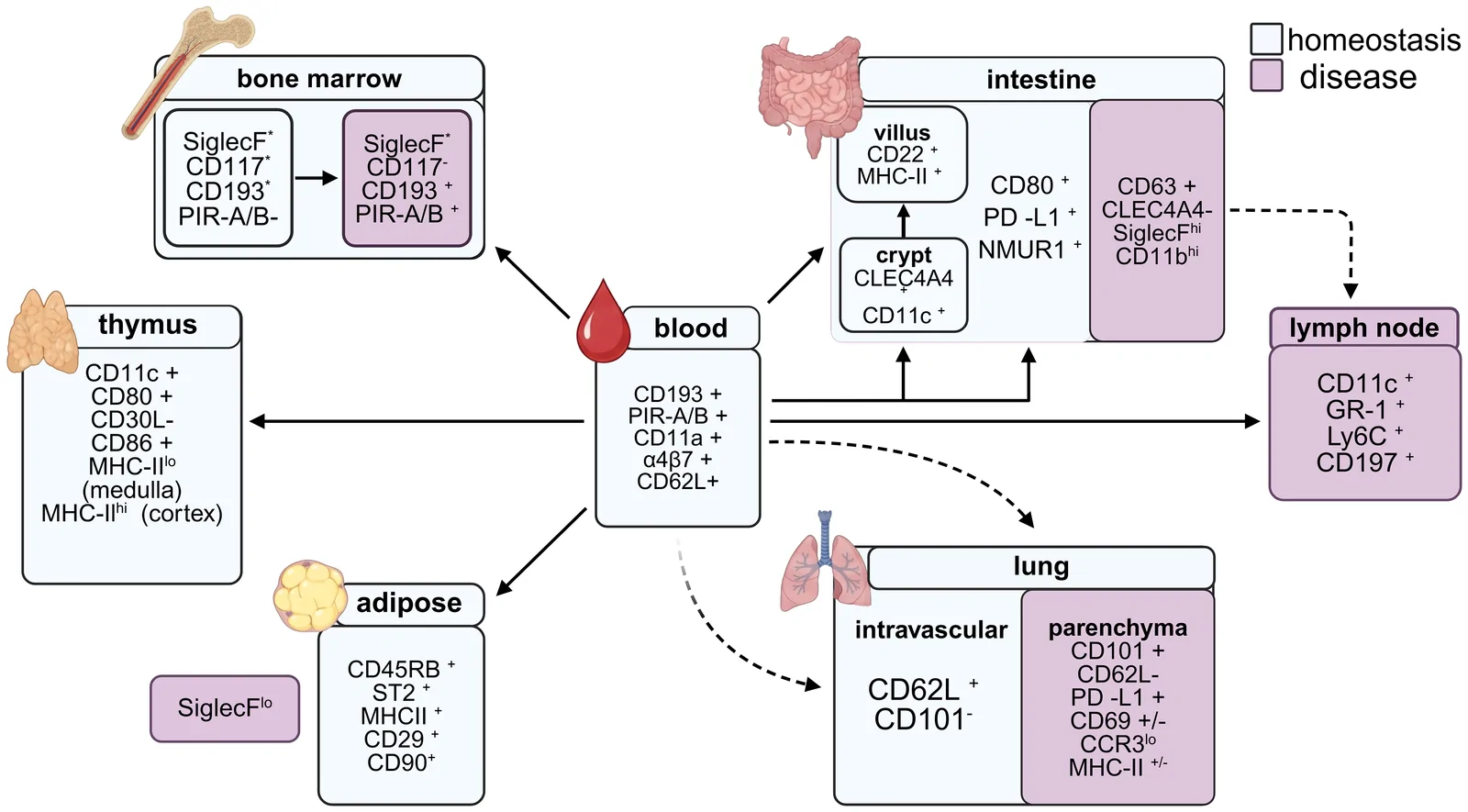
Candidate tissue- and disease-associated eosinophil phenotypes across anatomical compartments. Created in BioRender. Zhu, W. (2026) https://BioRender.com/exhgu74.

Representative eosinophil phenotypes differ across blood and tissue compartments and between homeostatic and inflammatory settings. Marker combinations shown in the schematic are study and species dependent; several derive from mouse models and are not standardized for human autoimmune disease. Created with .

Beyond the classical rEOS/iEOS dichotomy, recent research has further identified multiple novel functional subsets, including regulatory EOS, fibrotic EOS, and immune-activating EOS (27, 76). Regulatory EOS specifically secrete high levels of IL-10 and TGF-β, which can suppress the activation of autoreactive T and B cells, maintain peripheral immune tolerance, and inhibit autoimmune inflammation. Fibrotic EOS can continuously secrete pro-fibrotic mediators, inducing fibroblast proliferation and extracellular matrix deposition, mediating tissue remodelling and organ fibrosis. Immune-activating EOS can function as antigen-presenting cells, directly activating adaptive immune responses and amplifying autoimmune reactions (77, 78).

The plasticity of EOS is manifested by the dynamic interconversion between subsets based on microenvironmental signals. Inflammatory stimulation can induce the transition of homeostatic rEOS to pathogenic iEOS, and upon inflammation resolution and microenvironment repair, iEOS can revert back to the rEOS phenotype to participate in tissue repair (61). This dynamic plasticity is a feature distinguishing EOS from other terminally differentiated immune cells and also explains the cytological mechanisms underlying the fluctuating inflammation and repeated relapses characteristic of autoimmune disease courses. Additionally, EOS from different tissue sources possess unique tissue imprints. The transcriptomes and functional phenotypes of gut, airway, and skin EOS exhibit significant differences, leading to tissue-specific pathogenic mechanisms in different autoimmune diseases (61, 79). Eosinophil tissue adaptation is governed by cellular origin, inflammatory milieu, local cell-to-cell communication and tissue residence duration (**Figure 6**).

**Figure 6:**
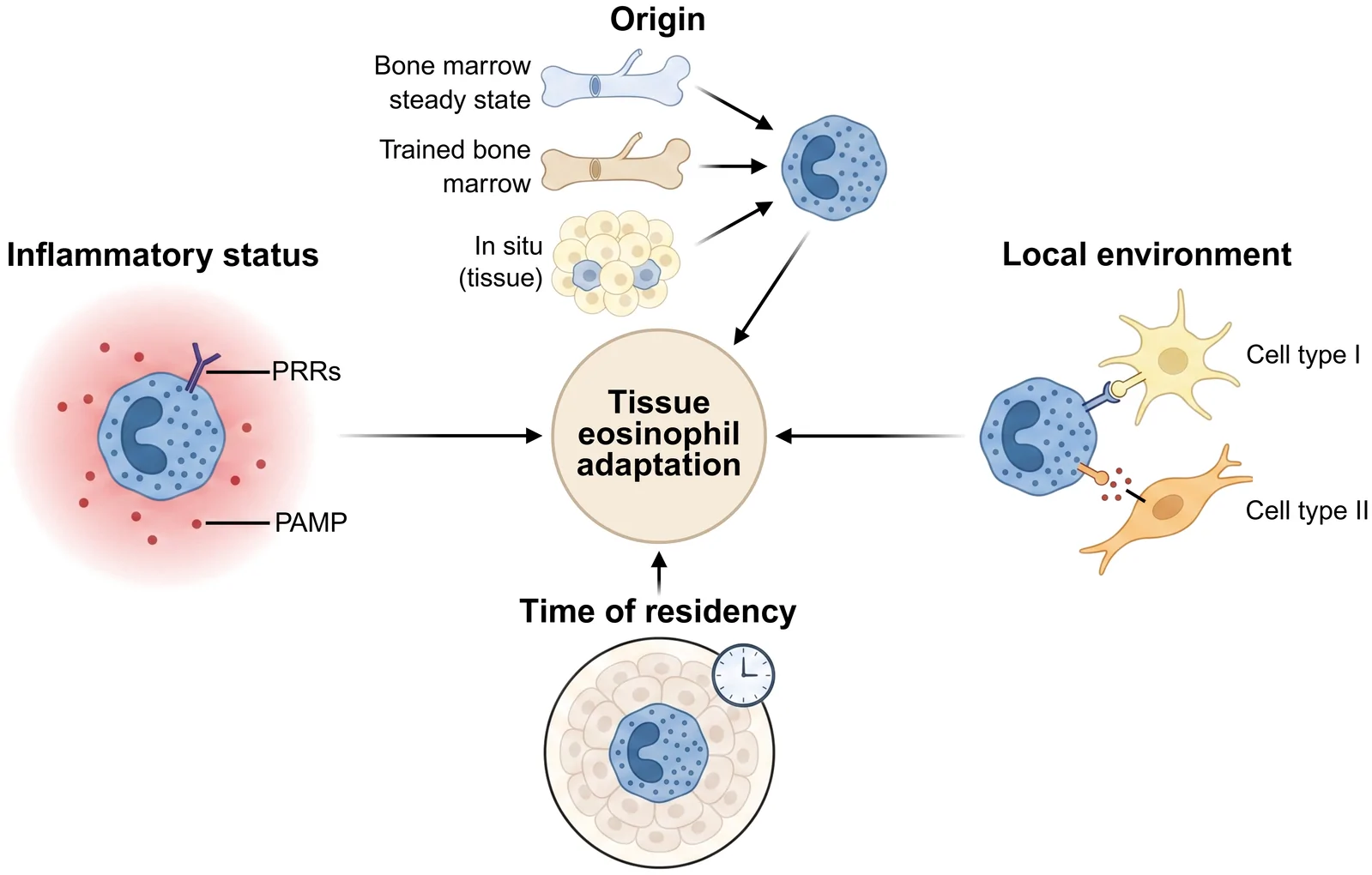
Major determinants of tissue eosinophil adaptation and functional heterogeneity. Created in BioRender. Zhu, W. (2026) https://BioRender.com/fqlb1jl.

Eosinophil state can be shaped by developmental origin, inflammatory signals, local cellular and metabolic cues, and duration of tissue residence. These determinants interact rather than defining fixed, mutually exclusive subsets. Created with .

### 2.6 Bidirectional eosinophil functions: a context-dependent model

EOS can contribute to cytotoxic injury through granule proteins, ROS, lipid mediators, cytokines, and EETs, while in other settings they support barrier integrity, tissue repair, metabolic homeostasis, and immune regulation. These functions are not mutually exclusive and can change with recruitment route, activation intensity, anatomical niche, and disease stage. The same mediator can also have different consequences across tissues; accordingly, cell number alone is an incomplete proxy for eosinophil function (11, 27, 39, 56).

For autoimmune disease, this framework implies three practical distinctions: (i) circulating eosinophilia versus tissue eosinophil activation, (ii) broad depletion versus state-selective modulation, and (iii) association with disease activity versus evidence that EOS are necessary or sufficient for pathology. These distinctions recur across the disease-specific sections below and are used to calibrate causal language and therapeutic inference. Furthermore, peripheral inflammatory signals reshape both local tissue eosinophil pools and bone marrow hematopoiesis to propagate systemic eosinophilic responses (**Figure 7**).

**Figure 7:**
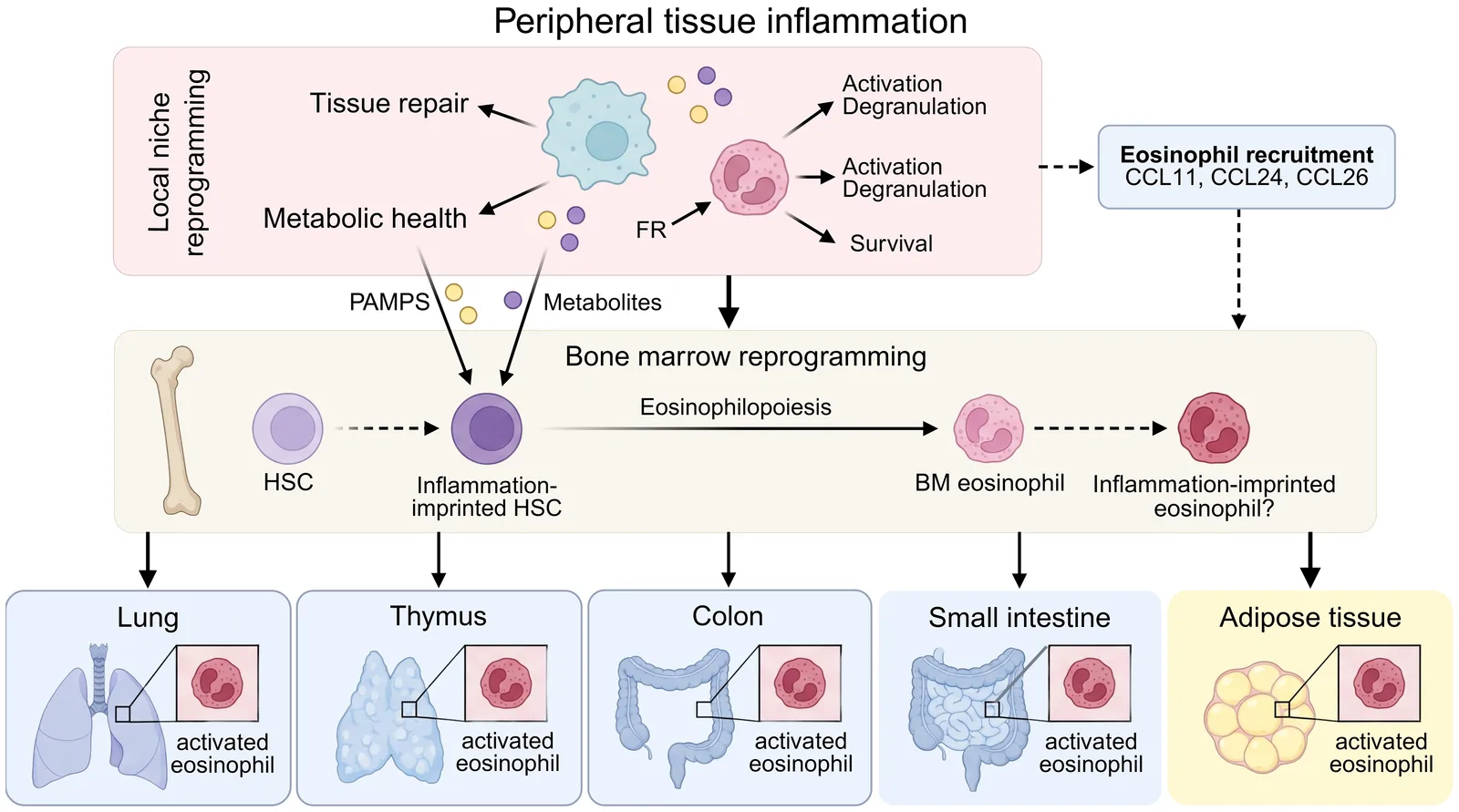
Local niche and bone marrow signals shaping eosinophil responses across tissues. Created in BioRender. Zhu, W. (2026) https://BioRender.com/5u1vfdq.

Local inflammatory cues can alter eosinophil activation, survival, degranulation, and repair functions while also influencing bone marrow eosinophilopoiesis and subsequent recruitment through eotaxin pathways. The model emphasizes multilevel reprogramming rather than a single linear pathway. Created with .

It is worth noting critically that the function of EOS is not a simple binary division of “beneficial/harmful” but rather exhibits dose-dependent, spatiotemporally dependent, and environment-dependent characteristics (56). Low-level, quiescent EOS exert homeostatic protective effects; moderately activated, locally infiltrated EOS initiate mild inflammatory responses and participate in damage repair; while excessively activated, massively accumulated EOS drive pathological inflammation and autoimmune injury. Concurrently, the function of EOS can undergo dynamic conversion at different stages of the disease course. They may participate in damage repair in the early disease stage but mediate fibrotic progression in the late stage. This spatiotemporal functional difference constitutes a core difficulty and controversy in current mechanistic research on eosinophil-associated diseases (27, 80).

### 2.7 Major unresolved questions

Three uncertainties limit translation. First, human eosinophil states lack a standardized, disease-spanning taxonomy. Second, most mechanistic studies do not establish whether eosinophil activation precedes autoimmunity or is recruited by an already inflamed tissue. Third, mouse and human EOS differ in receptor repertoire, tissue distribution, and granule biology, so preclinical subset and EETosis findings require explicit species annotation. These limitations are revisited in the Discussion and are incorporated into **Tables 1**–**4**.

**Table 2:** Proposed eosinophil phenotypes and candidate markers: functions, species, and evidence limitations.

Proposed state	Candidate markers/features	Functional tendency	Evidence base	Key caveat
Homeostatic/resident-like	CD62L+/CD101− (commonly cited scheme); tissue-residency programs	Homeostasis, barrier support, repair	Predominantly mouse; limited human tissue validation	Not a standardized human rEOS definition
Inflammatory/activated-like	CD62L−/CD101+ in mouse lung; activation-dependent CD11c in some datasets	Inflammatory mediator release, tissue injury	Mouse and selected human observational datasets	Phenotype varies by tissue and stimulus
Regulatory-like	PD-L1, CD80, and immunoregulatory programs reported in selected tissues	T-cell modulation; potential support of immune restraint	Preclinical and limited human tissue evidence	No consensus surface-marker panel
Neuromedin-responsive/tissue-adapted	NMUR1 reported in type 2 tissue circuits	Niche-dependent activation and neuroimmune cross talk	Mainly preclinical/tissue-context evidence	NMUR1 is not a universal EOS-subset marker
Siglec-defined inhibitory axis	Siglec-8 (human); Siglec-F (mouse)	Death/inhibitory signaling under defined conditions	Human *in vitro* for Siglec-8; mouse *in vivo*/*in vitro* for Siglec-F (81, 82)	Siglec-8 and Siglec-F are related but not equivalent
Intestinal active states	Transcriptional/tissue-adaptation programs rather than a single canonical marker	Host defence, inflammatory regulation, epithelial interaction	Single-cell/spatial and mouse/human comparative work (49)	State labels depend on dataset and anatomical compartment

**Table 3:** Cross-disease comparison of eosinophil roles and strength of evidence.

Disease context	Predominant role	Leading mechanisms	Candidate biomarkers	Evidence level	Main limitation
RA/inflammatory arthritis	Mixed; pathogenic and regulatory states proposed	Synovial recruitment; Treg–EOS cross talk; granule/ROS effects	Blood/tissue EOS; candidate rEOS signatures	MR + observational + preclinical	Human subset markers are not standardized; treatment evidence sparse
SLE	Context-dependent; generally adjunct rather than dominant	Tissue recruitment, degranulation, immune-cell cross talk	Blood EOS/ELR; tissue EOS in selected phenotypes	Small observational studies; limited mechanistic data	Organ/subtype heterogeneity; biomarker cut-offs not validated
EGPA/AAV	Strong pathogenic contribution in EGPA; uncertain adjunct role in GPA/MPA	IL-5–dependent expansion, degranulation, EETs, vascular/tissue injury	Blood EOS; eosinophil proteins; clinical phenotype	Human tissue + randomized trials in EGPA	Cannot extrapolate EGPA biology or treatment response to all AAV
CNS autoimmunity	Disease-specific and uncertain outside eosinophil-rich NMOSD lesions	Barrier trafficking, granule toxicity; model-dependent immune regulation	CSF/tissue EOS in selected settings	Human pathology + EAE/preclinical	EAE findings are not equivalent to human MS biology
Autoimmune endocrinopathies	Possible modulatory role	Islet/thyroid immune cross talk; type 2 signals	Peripheral EOS-derived indices	Mostly observational/preclinical	Direct tissue causality and therapeutic relevance unproven
Autoimmune dermatoses	Strong in bullous pemphigoid; variable in psoriasis/CTD skin	Degranulation, proteases, EETs, Fc/complement cross talk	Tissue EOS, ECP, CLC/galectin-10	Human pathology; disease-specific studies	Marked diagnostic and disease-stage heterogeneity
IBD	Bidirectional and compartment dependent	Barrier injury, ROS, immune cross talk, repair/fibrosis	Tissue EOS; granule proteins	Human observational + strong preclinical/tissue work	Inflammatory vs. protective states are difficult to separate
SSc/EF/morphea	Potential pro-fibrotic role; strongest eosinophilic signal in EF	Type 2/fibrotic mediators; stromal cross talk	Blood/tissue EOS in selected cohorts	Small cohorts and pathology series	Low sample size; temporal causality unresolved
Pediatric autoimmune disease	Insufficient age-specific evidence	Likely disease- and developmental-context dependent	No validated pediatric EOS biomarker panel	Sparse pediatric cohorts	Adult thresholds and subset schemes should not be extrapolated

**Table 4:** Comparative mechanism, evidence, and translational trade-offs of eosinophil-related biologic strategies.

Agent/strategy	Target	Effect on eosinophils	Highest relevant evidence	Safety uncertainty	Patient-selection/cost considerations
Mepolizumab	IL-5	Reduces production/survival; incomplete depletion	EGPA: randomized placebo-controlled benefit (23)	Generally tolerated; infection/malignancy excess not established; long-term physiological consequences of sustained suppression incompletely defined	High biologic cost; patient selection supported mainly in EGPA
Reslizumab	IL-5	Reduces blood EOS; not complete depletion	Robust asthma evidence; autoimmune evidence limited	IV administration; hypersensitivity/anaphylaxis concern; limited autoimmune long-term data	No established autoimmune cost-effectiveness evidence
Depemokimab	IL-5	Prolonged IL-5 neutralization; not proven subset-selective	Long-acting asthma program; autoimmune efficacy not established	Long-term deep pathway suppression requires disease-specific safety data	Convenience may improve dosing burden, but autoimmune value/cost remain unknown
Benralizumab	IL-5Rα	ADCC-mediated near-complete blood EOS depletion	EGPA: noninferior to mepolizumab in MANDARA (24)	Long-term consequences of near-complete depletion remain an evidence gap; no established malignancy signal	Treatment choice should consider phenotype and steroid-sparing goals, not depletion depth alone
CCR3/OXER1 inhibitors	Chemotaxis/activation receptors	Primarily blocks recruitment/activation rather than systemic depletion	Early/preclinical; no established autoimmune indication	Human efficacy and long-term safety insufficiently defined	Experimental; cost-effectiveness cannot yet be assessed
Dupilumab	IL-4Rα (IL-4/IL-13 signaling)	Does not directly deplete EOS; may transiently raise blood EOS	Established in type 2 diseases; not a general autoimmune EOS therapy	Rare eosinophilic complications/EGPA-like presentations; mechanism may include redistribution or unmasking rather than proven IL-5 compensation	Requires screening for pre-existing eosinophilic disease and clinical monitoring

## 3 Comparative evidence across autoimmune disease spectra

### 3.1 Mechanisms of eosinophil participation in the pathogenesis of inflammatory arthritis

To avoid an organ-by-organ catalogue, the disease sections are interpreted through the same four mechanistic axes: recruitment/tissue imprinting, direct effector injury, immune-network cross talk, and repair/fibrosis. **Table 3** provides a cross-disease comparison of predominant eosinophil roles, candidate biomarkers, and the principal weakness of the evidence. **Table 2** separately summarizes selected quantitative human findings so that mechanistic claims can be weighed against study design and effect size.

#### 3.1.1 Rheumatoid arthritis: genetically proxied risk, tissue heterogeneity, and an unresolved regulatory-eosinophil model

RA studies support eosinophil heterogeneity but do not yet define a stable human “regulatory eosinophil” lineage. Synovial and circulating EOS can display activation-dependent phenotypes, and experimental work suggests reciprocal interactions with regulatory T cells (Tregs). The frequently invoked rEOS/iEOS distinction should therefore be treated as a working model rather than an established clinical taxonomy; surface-marker definitions vary by species, tissue, and study platform (83–90).

Genetic evidence adds a population-level signal. A two-sample Mendelian randomization analysis using RA GWAS data from 14,361 cases and 43,923 controls included 368 eosinophil-count instrumental variants and reported an inverse-variance-weighted association between genetically predicted eosinophil count and RA risk (OR 1.58, 95% CI 1.39–2.68), with replication in an independent analysis and multiple sensitivity methods (20). These data strengthen the case for a relationship between eosinophil biology and RA susceptibility, but heterogeneity among instruments and the assumptions inherent to Mendelian randomization preclude inference of a direct eosinophil cellular mechanism.

Preclinical and translational studies further suggest a Treg–eosinophil regulatory circuit in arthritis. Hattori et al. reported that synovial ST2-expressing Tregs can suppress immunoregulatory eosinophil activity in experimental and translational settings (90). This finding is mechanistically provocative, but the phenotype of human regulatory EOS is not standardized and may not map directly onto murine CD62L/CD101-defined states. Accordingly, claims of an rEOS “phenotype switch” in RA should remain hypothesis-level until replicated with human tissue–resolved functional studies.

It is worth critically noting that current research on the bidirectional regulatory mechanisms of EOS in RA still has significant limitations. First, the specific surface markers for rEos have not been fully clarified. The lack of a precise sorting and identification system leads to heterogeneity in the cellular definition and functional characteristics of rEos across different studies, making it difficult to achieve standardized validation of results (85). Second, the upstream regulatory pathways governing eosinophil subtype conversion have not been elucidated. What microenvironmental signals drive the switch of EOS from a pathogenic phenotype to a regulatory phenotype, and what are the spatiotemporal dynamics of this conversion, remain core gaps in the field. Finally, existing research is mostly based on clinical sample observations and animal model validation, lacking large-sample prospective cohort studies to clarify the precise association between dynamic eosinophil changes and RA disease activity, prognosis, and treatment response, which limits their clinical translational application (83).

#### 3.1.2 Dual regulatory and homeostatic imbalance mechanisms of eosinophils in osteoarthritis

Osteoarthritis was previously defined as a degenerative joint disease. However, research in recent years has confirmed that low-grade chronic inflammation is a core driving factor in the occurrence and development of OA. Micro-inflammatory imbalances mediated by innate immune cells, cartilage degradation, and abnormal bone remodelling collectively drive the pathological process of OA (91, 92). Compared with RA, research on EOS in OA started later and was long neglected. Early studies only identified eosinophil infiltration in the joint microenvironment of OA patients but lacked systematic analysis of their functional mechanisms (91). Recent immune infiltration omics and functional experiments have confirmed that EOS are important regulatory cells in the OA joint immune microenvironment, possessing dual functions of both inflammatory activation and tissue protection. Their functional imbalance is an important trigger for the persistence of low-grade inflammation and the progression of cartilage degeneration in OA, presenting regulatory characteristics distinctly different from those in RA (93).

Under OA pathological conditions, aberrantly activated EOS primarily exert pro-inflammatory and pro-degenerative effects. Cartilage wear and tissue damage in OA joints can release large amounts of damage-associated molecular patterns (DAMPs), activating local innate immune responses, inducing the secretion of eosinophil-activating factors such as IL-5, and promoting the infiltration and activation of peripheral EOS into the joint synovium and cartilage tissue (94). Activated EOS can induce chondrocyte apoptosis and inhibit cartilage matrix synthesis by releasing oxidative stress mediators and pro-inflammatory cytokines. Simultaneously, they activate the pro-inflammatory phenotype of synovial fibroblasts, maintaining the state of low-grade chronic inflammation in the joint and accelerating cartilage degenerative lesions (93). Transcriptomic analysis has confirmed that the level of eosinophil infiltration in OA joint tissue is significantly positively correlated with the degree of cartilage degeneration and inflammation scores, and core genes such as FOS can participate in the process of OA immune dysregulation by regulating eosinophil infiltration (94). Furthermore, studies on metabolic inflammatory arthritis such as gouty arthritis have also confirmed that EOS can participate in crystal-induced joint inflammation by releasing extracellular traps, suggesting they also play a pro-inflammatory role in metabolism-related joint inflammation, providing new theoretical support for the metabolic inflammatory mechanisms in OA (95).

Simultaneously, EOS exert crucial protective functions in OA, primarily manifested in two aspects: maintaining bone homeostasis and regulating inflammation resolution. Under physiological conditions, EOS can precisely regulate the bone remodelling balance by secreting functional factors, specifically inhibiting the excessive activation of osteoclasts, reducing abnormal bone resorption, and maintaining the structural stability of joint bone tissue (68, 87). Animal model studies have confirmed that eosinophil-deficient mice exhibit significant bone loss and exacerbated bone destruction under inflammatory stimulation, confirming the bone-protective role of EOS (87). In the inflammatory resolution phase of OA, functional EOS can induce synovial fibroblasts to secrete lumican protein, activating the TGF-β signaling pathway, effectively inhibiting cartilage matrix degradation and alleviating cartilage damage, thus playing a joint-protective role (96). This dual function of “pro-inflammatory injury–anti-inflammatory protection” endows EOS with complex spatiotemporally dependent roles in OA: in the early disease stage, they primarily play anti-inflammatory and homeostatic maintenance roles; as inflammation persists and the microenvironment becomes dysregulated, the eosinophil phenotype undergoes an aberrant switch, gradually exerting pro-inflammatory and pro-degenerative effects, driving the progression of OA from physiological degeneration to pathological inflammation (93).

The controversy in current OA research lies in the functional specificity and disease relevance of EOS. Some clinical studies have found that eosinophil levels in the synovial fluid of healthy individuals are significantly higher than those in patients with temporomandibular joint OA, suggesting that eosinophil reduction may be associated with the onset of OA (96), while other studies have confirmed that increased eosinophil infiltration exacerbates OA inflammatory degeneration (94). The core reason for these contradictory conclusions lies in the heterogeneity of OA disease stages, lesion locations, and microenvironmental states, as well as the lack of standards for identifying eosinophil subtypes. Moreover, compared with macrophages and neutrophils, the infiltration abundance of EOS in OA is relatively low, leading to a long-term underestimation of their functional role. Their specific molecular pathways and cellular interaction mechanisms still require further exploration (93).

#### 3.1.3 Pathogenic features of eosinophils in other types of inflammatory arthritis

In addition to the two core subtypes of RA and OA, EOS are also widely involved in the pathogenesis of special types of inflammatory arthritis such as psoriatic arthritis and eosinophilic synovitis, exhibiting disease-specific pathogenic mechanisms and clinical features. Psoriatic arthritis is a chronic inflammatory joint disease associated with psoriasis, characterized by enthesitis, synovitis, and bone erosion. Th2-type inflammatory dysregulation and eosinophil activation are important immunological features (97). Studies have confirmed that eosinophil infiltration levels are significantly elevated in the peripheral blood and joint tissues of PsA patients, and eosinophil-related immune dysregulation is closely associated with complications like PsA uveitis and disease activity (97). Meanwhile, core type 2–associated cytokines such as IL-13 can participate in the pathogenesis of PsA by regulating eosinophil function, and gene-targeted inhibition of IL-13 can significantly regulate eosinophil-mediated inflammatory responses, reducing the risk of PsA (98). Gut microbiota dysbiosis can indirectly influence PsA disease progression by modulating eosinophil infiltration levels, further supporting the interpretation that EOS are a key hub cell in the multidimensional pathogenesis of PsA (99).

Eosinophilic synovitis is a rare specific inflammatory joint disease characterized by massive eosinophil infiltration of the joint synovium and sterile inflammation. Its clinical symptoms are easily confused with septic arthritis, leading to misdiagnosis and missed diagnosis (100). It is more common in children, primarily manifested as joint redness, swelling, pain, and limited mobility. Pathological biopsy reveals a large number of mature EOS specifically infiltrating the synovial tissue, with no evidence of bacterial infection (100). The pathogenesis of this disease is closely related to excessive type 2 immune activation and IL-5–mediated eosinophil proliferation and infiltration. Glucocorticoids and anti-IL-5 monoclonal antibodies can effectively suppress eosinophil activation and rapidly alleviate joint inflammation, supporting the interpretation that EOS are the core pathogenic effector cells of this disease (100). Furthermore, in paediatric inflammatory arthritis such as juvenile idiopathic arthritis, immunohistological studies have confirmed that abnormal eosinophil infiltration is an important marker of immune dysregulation, participating in the maintenance of chronic joint inflammation (101).

#### 3.1.4 Therapeutic targets and clinical translation controversies of eosinophil-targeted therapies in inflammatory arthritis

Based on the core regulatory role of EOS in inflammatory arthritis, biologics targeting EOS and their upstream regulatory pathways have become a research hotspot for novel therapeutic strategies in inflammatory arthritis. Targeted drugs represented by the anti-IL-5 monoclonal antibody mepolizumab have shown good efficacy in eosinophil-associated autoimmune vasculitis and arthritis (102, 103). Clinical guidelines explicitly recommend the preferential use of mepolizumab for patients with relapsed, refractory eosinophilic granulomatosis with polyangiitis (EGPA) complicated by joint inflammation, as it can significantly reduce peripheral blood and tissue eosinophil levels, suppress joint inflammation, decrease the disease relapse rate, and reduce the dosage of glucocorticoids (102, 104). Long-term follow-up studies have confirmed that mepolizumab can achieve long-term remission in EGPA patients with good safety and tolerability, providing a mature targeted treatment option for eosinophil-mediated inflammatory joint diseases (105, 106). In addition, IL-5 receptor antagonists such as benralizumab can effectively control refractory eosinophilic autoimmune inflammation by depleting activated EOS, offering new drug choices for targeted arthritis therapy (104).

However, the clinical translation of eosinophil-targeted therapy for inflammatory arthritis still faces significant controversies and bottlenecks. First, due to the bidirectional regulatory role of EOS in RA and OA, indiscriminate inhibition of EOS may disrupt their anti-inflammatory protective functions, leading to bone homeostasis imbalance, impaired inflammatory resolution, and even exacerbation of joint damage. This is the core reason for the poor efficacy of broad-spectrum anti-eosinophil drugs in common RA and OA (87, 90). Second, existing targeted drugs lack cellular subtype specificity, unable to precisely inhibit pathogenic EOS while preserving the protective functions of regulatory EOS. Insufficient targeting precision limits their clinical application scenarios (83). Finally, current clinical research is mostly focused on rare eosinophilic autoimmune diseases like EGPA. There is a severe lack of large-sample, randomized controlled clinical trials for common inflammatory arthritis such as RA, OA, and PsA. Core issues such as drug efficacy, applicable populations, and optimal intervention timing remain undefined (104).

#### 3.1.5 Evidence synthesis for inflammatory arthritis

Across inflammatory arthritis, EOS are better supported as context-dependent modifiers than as uniformly dominant effector cells. RA has genetic and mechanistic signals, but human subset definitions and interventional evidence remain limited; OA and PsA evidence is more indirect and should not be used to infer autoimmune causality. Future studies should pair tissue-resolved phenotyping with longitudinal sampling and prespecified clinical outcomes rather than relying on peripheral eosinophil counts alone.

### 3.2 Mechanisms of eosinophil participation in the pathogenesis of systemic lupus erythematosus

SLE is a heterogeneous systemic autoimmune disease dominated by loss of self-tolerance, autoantibody formation, immune-complex injury, and type I interferon-associated inflammation. Eosinophil involvement is less consistent and appears concentrated in selected tissue phenotypes rather than across SLE as a whole. The evidence below therefore distinguishes peripheral blood changes from tissue infiltration and avoids treating EOS as a universal SLE effector population (107–109).

#### 3.2.1 Pathological distribution heterogeneity and characteristic phenotypes of eosinophils in systemic lupus erythematosus

The infiltration and activation levels of EOS in the peripheral blood and affected tissues of SLE do not follow a uniform pattern but rather exhibit strict disease subtype dependency, organ specificity, and disease course variability. This is the core reason for the contradictory conclusions drawn from previous studies, such as “EOS is unrelated to SLE pathogenesis” and “EOS participates in SLE injury” (110, 111). Recent histopathological studies have confirmed that the distribution characteristics of EOS in SLE-affected organs like the skin, kidneys, serosa, and subcutaneous fat exhibit significant differentiation, and there are clear boundaries in the EOS infiltration patterns among different clinical subtypes. This challenges the traditional one-sided perception that “SLE is dominated by neutrophil and lymphocyte infiltration, and EOS are rare” (112, 113).

In cutaneous lupus subtypes, EOS infiltration exhibits highly specific negative/positive differentiation. In the lesional skin of discoid lupus erythematosus (DLE) and subacute cutaneous lupus erythematosus (SCLE), EOS are almost completely absent or only very sparsely distributed. This feature has been confirmed by multiple systematic studies and can serve as a key differential pathological indicator for distinguishing cutaneous lupus from other eosinophilic inflammatory skin diseases (110, 111). In contrast, significant EOS infiltration is enriched in lupus profundus (lupus panniculitis), bullous systemic lupus erythematosus (BSLE), and drug-induced lupus skin lesions. A classic study by Peters et al. showed that 24% of 33 patients with lupus panniculitis exhibited specific EOS infiltration in subcutaneous fat tissue, with some severe cases showing massive, aggregated EOS infiltration, and the degree of infiltration positively correlated with the extent of subcutaneous fat necrosis and fibrosis (112). Although BSLE lesions are dominated by neutrophil infiltration, activated EOS and degranulation products can be detected in active lesions and subepidermal blister fluid, acting as important auxiliary factors mediating epidermal basement membrane injury (114). Additionally, in cases of drug-induced lupus serositis induced by biologics like adalimumab, pleural biopsy tissue reveals a large number of EOS, acute inflammatory cells, and lymphoid follicle aggregates, supporting the interpretation that lupus inflammation induced by exogenous interventions can specifically activate the EOS pathway (113).

Peripheral eosinophil-based indices have been explored as low-cost activity markers in SLE, but results are cohort dependent and should not be generalized to lupus nephritis. In a 2025 cross-sectional study, lower eosinophil-to-lymphocyte ratio (ELR) was associated with flare: an ELR threshold ≤0.0076 distinguished flare from remission with AUC 0.647, whereas ≤0.029 distinguished flare from healthy controls with AUC 0.775 (21). These thresholds require external validation and should not be presented as universal cut-offs or kidney-specific biomarkers.

From the perspective of cellular phenotypic characteristics, EOS in the SLE microenvironment present a unique activation phenotype distinct from that seen in allergic diseases and EGPA. While classical type 2 inflammation-induced EOS are characterized by high IL-5 receptor expression, persistent degranulation, and potent pro-inflammatory effects, EOS in SLE are in a state of “low-grade activation, persistent stress,” possessing the dual characteristics of pro-inflammatory mediator secretion and immunomodulatory molecule expression (115, 116). Transcriptomic studies have confirmed that EOS derived from SLE patients highly express specific functional genes such as PRG2 and RNASE2. The eosinophil granule protein encoded by PRG2 can participate in neuroinflammation and autoimmune regulation and is closely related to the pathogenesis of neuropsychiatric lupus (NPSLE) (117). Simultaneously, SLE-EOS can secrete low levels of Th2 cytokines like IL-4 and IL-13 while responding to the high concentration of IFN-I stimulation in the SLE microenvironment, forming a unique phenotype of mixed Th1/Th2 activation. This is also a core molecular feature distinguishing them from purely eosinophilic inflammation (117, 118).

#### 3.2.2 Core molecular and immune mechanisms of eosinophil participation in SLE pathogenesis

EOS are not passive bystander cells in SLE pathogenesis but deeply participate in the initiation, sustained progression, and organ-specific damage of SLE through multiple pathways, including degranulation-mediated tissue injury, regulation of adaptive immunity, participation in immune complex deposition, and mediation of epithelial barrier damage. Their mechanisms can be divided into four major interactive pathways, each forming a complex positive feedback regulatory network with the classical SLE IFN-I pathway and B-cell activation pathway (119, 120).

##### 3.2.2.1 Degranulation-mediated direct tissue toxicity injury pathway

The core effector function of EOS depends on the release of specific granule proteins, including ECP, MBP, and eosinophil peroxidase (EPO). These toxic proteins can directly damage target organ cells and disrupt tissue structural integrity, serving as a direct effector mechanism of multi-organ injury in SLE (109, 110). In LN, ECP released by activated EOS infiltrating the renal tubulointerstitium can directly damage renal tubular epithelial cells, induce apoptosis and interstitial fibrosis, and simultaneously disrupt podocyte homeostasis, exacerbating proteinuria and renal function impairment (121). Recent mechanistic studies have confirmed that ECP not only has direct cytotoxicity but can also remotely regulate immune responses through the exosomal pathway: aberrant overexpression of ECP in T cells and exosomes can induce upregulation of IFN-γ secretion, promoting the proliferation and differentiation of follicular helper T cells (Tfh) and plasma cells, thereby driving the massive production of autoantibodies and forming a positive feedback loop of “EOS degranulation–inflammation amplification–autoimmune activation” (117). In skin and subcutaneous tissue injury, EOS granule proteins can induce adipocyte necrosis, degradation of dermal collagen, and vascular endothelial injury, acting as core mediating factors in the subcutaneous nodules, ulcers, and fibrosis of lupus panniculitis (112). Unlike acute necrotic inflammation mediated by neutrophils, EOS-mediated tissue injury in SLE is characterized by chronic, persistent, and low-grade features, lacking massive neutrophil infiltration, with interstitial chronic inflammation and fibrosis as the main pathological manifestations. This also explains the clinical features of lupus panniculitis, which is slow to resolve and prone to scarring (120). Furthermore, in patients with SLE complicated by serositis, EOS degranulation products in pleural and pericardial tissues can induce increased serosal capillary permeability and inflammatory exudation, triggering eosinophilic serosal effusions, representing a special pathological phenotype of serosal involvement in SLE (113).

##### 3.2.2.2 Innate immune pathway aberrant activation and barrier injury mechanisms

Epithelial barrier functional deficiency is one of the initiating factors for autoimmune diseases. Damage to the skin, respiratory tract, and renal epithelial barriers can lead to the exposure of self-antigens, triggering subsequent autoimmune cascades (119). EOS can participate in SLE epithelial barrier injury by regulating epithelial cell apoptosis and the degradation of tight junction proteins. Studies have confirmed that inflammatory mediators secreted by activated EOS can disrupt the tight junctions of skin and renal tubular epithelial cells, increase tissue permeability, promote the continuous release of self-antigens such as nuclear and cytoplasmic antigens, and break autoimmune tolerance (119). Simultaneously, the IL-33/ST2 axis is aberrantly activated in the SLE microenvironment. As a key epithelium-derived alarmin, IL-33 can specifically bind to the ST2 receptor on the EOS surface, promoting EOS activation, proliferation, and chemotactic aggregation, further amplifying local inflammatory responses and forming a vicious cycle between barrier injury and EOS activation (122).

Additionally, EOS can participate in innate immune dysregulation in SLE through pattern recognition receptors. EOS express various Toll-like receptors (TLRs) on their surface, which can recognize nucleic acid-containing self-antigens abundantly present in SLE patients, activating the downstream NF-κB inflammatory pathway, inducing the secretion of pro-inflammatory cytokines and chemokines, and recruiting more inflammatory cell infiltration (110). Meanwhile, EOS can regulate the balance of type I and type II interferons: on one hand, they respond to the classical SLE IFN-I inflammatory microenvironment, maintaining their own activation status; on the other hand, they secrete factors that induce upregulation of IFN-γ expression, disrupting the Th1/Th2 immune balance and exacerbating systemic immune dysregulation (117).

##### 3.2.2.3 Adaptive immune regulation and autoantibody production mechanisms

In traditional understanding, EOS were considered merely terminal effector cells. However, recent research has confirmed that they possess potent antigen-presenting and immunoregulatory functions and can directly participate in the core links of adaptive immune dysregulation in SLE (110). Activated EOS can express MHC class II molecules and co-stimulatory molecules (CD80/CD86), directly presenting self-antigens to CD4^+^ T cells, promoting the activation and proliferation of autoreactive T cells, and breaking central and peripheral immune tolerance (109). Meanwhile, EOS can regulate the differentiation of T-cell subsets by secreting cytokines: their secreted IL-4 can inhibit excessive activation of Th1 and Th17 cells while promoting Th2 cell polarization. Hyperactivity of Th2-type immunity is an important trigger for humoral immune dysregulation and autoantibody production in SLE (117).

At the level of B-cell regulation, EOS can promote autoantibody generation and plasma cell survival through indirect pathways, but this mechanism exhibits significant context dependency and is a controversy in current research. An animal experimental study by Haberland et al. found that in lupus model mice, EOS were not essential cells for the maintenance of bone marrow plasma cells or the sustained production of autoantibodies. Knockout of EOS did not significantly reduce autoantibody levels or delay the onset of lupus, suggesting that EOS do not participate in the humoral immune regulation during the disease maintenance phase of SLE (108). However, multiple clinical studies have reached opposite conclusions: in patients with active SLE, activated EOS can promote the proliferation and differentiation of B cells into plasma cells by secreting inflammatory factors and regulating Tfh cell function, increasing the titers of autoantibodies such as anti-dsDNA and antinuclear antibodies, participating in immune activation during the acute phase of the disease (117, 119). The above contradictory results suggest that the regulation of adaptive immunity in SLE by EOS is disease-course-dependent: in the disease initiation phase, EOS participate in the breakdown of immune tolerance and the initial production of autoantibodies; in the disease maintenance phase, the regulatory role of EOS can be compensated by other immune pathways and is not essential. This bidirectional regulatory pattern is an important component of the immune heterogeneity in SLE.

##### 3.2.2.4 Chemokine-mediated targeted EOS infiltration mechanism

The specific accumulation of EOS in SLE-affected tissues depends on the aberrant activation of the chemokine-receptor axis, among which the CXCL10/CXCR3 and eotaxin/CCR3 pathways are the core regulatory pathways (117, 123). CXCL10 levels are significantly elevated in the serum and affected tissues of SLE patients. This chemokine can specifically bind to the CXCR3 receptor on the EOS surface, mediating the targeted infiltration of EOS into inflammatory sites such as the skin, kidneys, and serosa (123). Meanwhile, increased secretion of Th2-type chemokines such as IL-5 and eotaxin in the SLE microenvironment further promotes EOS bone marrow mobilization, peripheral activation, and tissue residency, forming a local EOS inflammatory microenvironment (109). Notably, the activation levels of the CXCL10 and IL-5 pathways are significantly reduced in DLE and SCLE lesions, unable to mediate effective EOS infiltration. This explains the pathological feature of EOS absence in these subtypes from a molecular level, supporting the interpretation that the subtype-specific activation of chemotactic pathways is a mechanism underlying the heterogeneity of EOS distribution in SLE (111, 124).

#### 3.2.3 Clinicopathological correlates of eosinophil-mediated multi-organ-specific injury in SLE

Based on the molecular mechanisms described above, aberrant activation and infiltration of EOS can specifically mediate multi-organ injury in SLE, including the skin, kidneys, serosa, and cardiovascular system. Moreover, their injury patterns exhibit clear organ specificity, highly consistent with the different clinical phenotypes of SLE, providing novel pathological evidence for disease stratification and prognosis assessment.

- At the level of skin injury: EOS only participate in the pathological damage of deep, bullous, and drug-induced lupus, not in the course of superficial cutaneous lupus. The chronic inflammation and fibrosis of subcutaneous fat mediated by EOS infiltration in lupus panniculitis are core pathological features distinguishing it from common lupus erythematosus and erythema nodosum, and also the key mechanism underlying the skin atrophy and scarring prone to occur in this disease (112, 120). In BSLE, EOS degranulation products assist neutrophils in disrupting the epidermal basement membrane, promoting subepidermal blister formation and exacerbating skin erosion and exudation (114). In contrast, due to chemotactic pathway inactivation and EOS absence, the inflammation in DLE and SCLE is predominantly lymphocyte-mediated interface dermatitis, without eosinophilic inflammatory involvement (110, 111).
- At the level of kidney injury: EOS infiltration is a specific pathological marker of active LN and is closely associated with poor prognosis. Urinary EOS count and ECP levels in LN patients can accurately reflect the degree of renal tubulointerstitial inflammation, independent of traditional indicators such as anti-dsDNA antibodies and complement C3/C4, and can serve as novel non-invasive biomarkers of LN activity (118, 121). LN patients with massive interstitial EOS infiltration have a significantly higher risk of renal tubular fibrosis and deterioration of renal function, representing an independent risk factor for progression to end-stage renal disease (121). Unlike the acute necrotizing glomerular injury in ANCA-associated vasculitis, EOS-mediated LN injury is characterized by chronic interstitial inflammation, podocyte injury, and interstitial fibrosis, with a more insidious pathological process and poorer prognosis (118, 125).
- At the level of serosal and cardiovascular injury: EOS participate in the pathological process of SLE serositis and myocarditis. EOS infiltration and degranulation in the pleura and pericardium of patients with SLE-associated eosinophilic serositis can induce serosal thickening, adhesions, and inflammatory effusions, acting as an important trigger for refractory lupus serositis (113). Simultaneously, chronic inflammation mediated by EOS activation can participate in the pathogenesis of SLE myocarditis. The toxic proteins they release can damage cardiomyocytes, inducing myocardial interstitial inflammation and fibrosis, representing a potential mechanism for occult cardiac injury in SLE (126, 127). Moreover, acute eosinophilic pneumonia and myocardial injury occurring in patients with drug-induced lupus are also closely related to excessive EOS activation (128).
- At the level of neuropsychiatric injury: The aberrant expression of the EOS-specific functional gene PRG2 is closely related to neuroinflammation and brain tissue injury in NPSLE. PRG2-mediated EOS-derived immune dysregulation can lead to increased cerebral vascular permeability and neuronal inflammatory damage, providing a new explanation for the occult pathogenesis of NPSLE (117).

#### 3.2.4 Evidence limitations in SLE

The SLE literature is heterogeneous in organ phenotype, treatment exposure, sampling compartment, and eosinophil definition. Most studies are observational, and tissue eosinophilia in selected cutaneous, renal, or serosal phenotypes does not establish that EOS initiate systemic autoimmunity. Apparent protective and pathogenic signals may reflect different disease stages or local niches. Until longitudinal human tissue studies establish temporal sequence and function, EOS should be considered potential modifiers and biomarkers in selected SLE endotypes rather than universal drivers.

### 3.3 Mechanisms of eosinophil participation in the pathogenesis of autoimmune vasculitis

Antineutrophil cytoplasmic antibody-associated vasculitis (AAV) includes EGPA, granulomatosis with polyangiitis (GPA), and microscopic polyangiitis (MPA), but eosinophil biology is not equivalent across these disorders. EGPA combines eosinophilic inflammation with variable ANCA-associated vasculitis and provides the clearest autoimmune setting in which EOS are both biologically prominent and therapeutically actionable. In GPA and MPA, neutrophil–ANCA mechanisms remain dominant and eosinophil findings should be interpreted as secondary or phenotype-modifying unless stronger evidence emerges (129–136).

#### 3.3.1 Infiltration characteristics and pathological localization differences of eosinophils in AAV subtypes

Tissue infiltration and aberrant activation of EOS are core pathological markers distinguishing EGPA from GPA and MPA and also the key cytological basis for the differentiation of inflammatory phenotypes among AAV subtypes (129, 134). The 2022 ACR/EULAR updated classification criteria for EGPA explicitly include significantly elevated peripheral blood EOS (>1,000 cells/μl) and diffuse tissue eosinophil infiltration as core diagnostic indicators, highlighting the specific pathological status of EOS in this disease (137, 138). In stark contrast to EGPA, classical GPA and MPA are dominated by neutrophil and lymphocyte infiltration. Previous research considered that EOS were not involved in these two subtypes. However, recent refined pathological studies have confirmed the presence of low-level, localized eosinophil infiltration in the acute-phase vascular injury sites of GPA and MPA. Moreover, the abundance of this infiltration is positively correlated with the degree of vascular necrosis and organ damage prognosis, suggesting that EOS also play a potential auxiliary pathogenic role in non-EGPA AAV, breaking the traditional dogma that “eosinophils specifically participate only in EGPA” (130).

In the pathological process of EGPA, eosinophil infiltration exhibits temporality and spatial specificity, forming a three-stage pathological mode of “airway prodromal inflammation—systemic vascular infiltration—granulomatous remodelling” (139). In the prodromal phase (asthma, chronic rhinosinusitis with nasal polyps stage), the local Th2-type inflammatory microenvironment in the airway mucosa drives the *in situ* proliferation and activation of EOS, releasing small amounts of toxic granule proteins and inducing chronic airway inflammation and epithelial damage, laying an inflammatory foundation for subsequent systemic vascular autoimmune activation (140). During the active disease phase, activated EOS break through the local mucosal barrier, enter the bloodstream, and migrate to the walls of small and medium-sized vessels throughout the body, affecting multi-organ vascular tissues such as the skin, lungs, kidneys, peripheral nerves, and cardiovascular system. This results in characteristic full-thickness eosinophil infiltration of the vessel wall, endothelial swelling, fibrinoid necrosis, and perivascular granuloma formation (141, 142). In the end-stage injury phase, persistently activated EOS, through chronic inflammatory stimulation and tissue remodelling, induce target organ fibrosis, vascular stenosis, and irreversible functional impairment, serving as a key driving factor for the poor long-term prognosis of EGPA (143).

From the perspective of pathological phenotype stratification, EGPA can be divided into two major subgroups based on ANCA status, and the pathogenic patterns of EOS show significant differentiation: MPO-ANCA-positive EGPA is dominated by a vasculitic phenotype, where EOS mainly infiltrate vessel walls, mediating acute vascular necrosis and systemic vascular inflammation; ANCA-negative EGPA is centered on purely eosinophilic inflammation and organ infiltration, with a milder degree of vascular injury, characterized mainly by eosinophil accumulation in soft tissues and parenchymal organs and granulomatous hyperplasia (24, 144, 145). This heterogeneity indicates that the pathogenic function of EOS does not solely rely on an increase in cell numbers but forms a synergistic regulatory network with the ANCA autoimmune pathway and local inflammatory microenvironment, ultimately determining the clinical phenotype and severity of the disease (146).

#### 3.3.2 Core effector mechanisms of eosinophil-mediated vascular injury

Eosinophil-induced vascular injury is not simply a single cytotoxic effect but occurs through four pathways—degranulation and release of toxic mediators, programmed death via EETosis, regulation of coagulation and inflammation, and remodelling of the immune microenvironment. These pathways mediate vascular endothelial injury, vessel wall destruction, inflammation amplification, and tissue remodelling, forming the core pathological chain of autoimmune vasculitis, with synergistic cascade amplification effects existing between pathways (131, 147, 148).

- Direct vascular endothelial injury mediated by granule toxic protein release: this is the most classical and core pathogenic mechanism of EOS. Activated EOS can release four types of specific toxic granule proteins: MBP, eosinophil cationic protein (ECP), eosinophil peroxidase (EPO), and eosinophil-derived neurotoxin (EDN) (147, 148). Among these, MBP and ECP carry strong positive charges and can directly bind to negatively charged phospholipids on the vascular endothelial cell membrane surface. This disrupts endothelial cell integrity, induces endothelial cell apoptosis and shedding, leading to vascular endothelial barrier disruption, causing increased vascular permeability, local oedema, and inflammatory cell infiltration (131). EPO can catalyze the reaction of local hydrogen peroxide with halide ions to generate highly toxic oxidative products, exacerbating oxidative stress injury in the vessel wall and inducing fibrinoid necrosis. EDN can damage perivascular nerve endings and parenchymal cells, mediating the characteristic peripheral neuropathy and multi-organ damage of EGPA (149). Notably, these toxic proteins can dissociate from EOS and deposit *in situ* on vessel walls and within tissue interstitium. Even during phases of eosinophil count decline and clinical remission, residual granule proteins can continuously induce chronic inflammation and tissue remodelling, explaining the pathological mechanism of persistent organ damage and high relapse rate during EGPA remission (150). Furthermore, compared with neutrophil-derived toxic mediators, eosinophil granule proteins have more persistent and specific tissue toxicity, serving as a core molecular feature distinguishing EGPA vascular injury from other AAV subtypes (133).
- EETosis-mediated amplification of vascular inflammation and immunothrombosis independent of cell death: this is a novel, key mechanism discovered in recent years, greatly improving the pathogenesis system of AAV. Traditional understanding held that extracellular trap-induced injury in vasculitis was solely mediated by neutrophil NETosis. However, the latest research confirms that a large number of EET structures can be detected in the vascular lesion tissues of patients with active EGPA, and their abundance is significantly positively correlated with disease activity (BVAS score) and the degree of vascular necrosis (133, 151). EETosis is a unique programmed inflammatory death mode of EOS. Activated EOS release extracellular reticular structures composed of DNA, histones, and granular toxic proteins. On one hand, these EETs can directly adhere to and damage vascular endothelial cells, activating the local complement system and amplifying local vascular inflammatory reactions. On the other hand, EETs can serve as a reservoir of autoantigens, exposing hidden intracellular antigens, breaking autoimmune tolerance, and inducing the production and affinity maturation of ANCA. This forms a positive feedback loop of “eosinophil activation—EETs release—self-antigen exposure—ANCA generation—further immune activation” (130, 133). Moreover, EETs can activate coagulation factor XII, initiating the endogenous coagulation pathway and inducing local microthrombus formation in blood vessels, thereby aggravating vascular ischaemic injury and mediating the characteristic immunothrombotic pathological changes of EGPA. This mechanism is also an important trigger for cardiovascular damage and organ infarction in EGPA (151, 152). Compared with NETosis, the pro-fibrotic and pro-thrombotic effects of EETs are stronger, making them unique drivers of chronic vascular remodelling in EGPA (151).
- Paracrine regulation by lipid inflammatory mediators and cytokines amplifies systemic autoimmune inflammation: activated EOS are not merely effector cells but also important immunoregulatory cells. They can synthesize and release lipid mediators such as leukotriene B4 and prostaglandin E2, as well as type 2–associated cytokines like IL-4, IL-5, and IL-13 (153, 154). Among these, IL-5 is the core regulatory factor for eosinophil proliferation, activation, and survival. It can maintain the sustained activation state of EOS through an autocrine pathway and inhibit their apoptosis, leading to abnormal eosinophil accumulation in tissues (155). IL-4 and IL-13 can induce vascular endothelial cell activation and high expression of adhesion molecules, promoting the recruitment of neutrophils, lymphocytes, and more EOS to the vessel wall, amplifying local inflammatory infiltration (153). Simultaneously, these type 2–associated cytokines can remodel the overall immune microenvironment of the body, disrupt the Th1/Th2 immune balance, inhibit the function of immunoregulatory cells, maintain systemic autoimmune dysregulation, and provide an immune basis for the sustained progression and relapse of vasculitis (154). Critically, previous research has overemphasized the auxiliary role of Th2 inflammation. The latest genomic studies confirm that Th2 inflammation in EGPA is not simply allergic inflammation but a pathological inflammation deeply coupled with autoimmune pathways. The vascular injury it mediates has autoimmune specificity, distinguishing it from common allergic eosinophilic inflammation (24).
- Immune cell interactive regulation mediates the pathological cascade of vasculitis: EOS can form a multidimensional interactive network with the core pathogenic cells of AAV (neutrophils, T cells, B cells, macrophages), amplifying autoimmune pathological damage (153, 156). First, EOS can promote neutrophil activation and NETosis release by releasing inflammatory mediators, enhancing ANCA-mediated neutrophil cytotoxicity and synergistically aggravating vascular wall injury. This explains the pathological feature of dual activation of both neutrophils and EOS in some EGPA patients (130). Second, EOS can regulate CD4^+^ T cell differentiation, promoting Th2 cell proliferation and inhibiting Treg cell function, weakening the body’s autoimmune tolerance capacity (154). Third, activated EOS can stimulate B-cell proliferation and differentiation, promoting the production of autoantibodies such as ANCA, directly driving the autoimmune response process (24). Fourth, EOS can induce macrophage polarization towards the M2 type. Excessive activation of M2 macrophages can mediate fibrotic remodelling of vessel walls and visceral tissues, leading to irreversible long-term organ damage in EGPA (157). This complex immune interactive network elevates EOS from single effector cells to core regulatory nodes in AAV immune dysregulation.

#### 3.3.3 Disease-specific pathogenic weight across AAV

The contribution of EOS differs sharply across AAV. In EGPA, eosinophilic inflammation is a defining biological component and human interventional data support therapeutic targeting of the IL-5 axis. In GPA and MPA, by contrast, neutrophil/ANCA biology remains dominant; reports of eosinophilia or tissue EOS are insufficient to establish an independent eosinophil-driven mechanism. The review therefore avoids extrapolating EGPA-specific mechanistic or therapeutic conclusions to other AAV subtypes (24, 129–134, 137–146).

Within EGPA itself, ANCA status is associated with different clinical patterns: ANCA-positive disease more often displays vasculitic manifestations, whereas ANCA-negative disease more often shows eosinophilic tissue infiltration. These are probabilistic phenotypes rather than mutually exclusive mechanisms, and both eosinophil-mediated and ANCA-associated pathways can coexist (24, 130, 144–146).

This heterogeneity is clinically important because blood eosinophil count, ANCA status, organ involvement, and treatment exposure capture different dimensions of disease. Mechanistic models should therefore be stratified by phenotype and sampling compartment rather than assuming one eosinophil program across EGPA.

#### 3.3.4 Unresolved questions and dupilumab-associated eosinophilia

Major uncertainties include the initiating signals for pathological eosinophil activation, the temporal relationship between eosinophilic inflammation and ANCA-associated immunity, and the lack of validated markers that distinguish pathogenic from homeostatic eosinophil states. These gaps are especially important when interpreting treatment-associated eosinophilia.

Transient peripheral eosinophilia and occasional eosinophilic complications, including EGPA-like presentations, have been reported during dupilumab therapy. The mechanism is not established in humans. Plausible explanations include altered tissue trafficking after IL-4Rα blockade, persistence of IL-5–driven eosinophil production, redistribution of circulating cells, and unmasking of previously subclinical eosinophilic asthma or EGPA. A compensatory IL-5/transcriptional-reprogramming model should therefore be presented as a hypothesis rather than the “proposed explanation” of these events (158, 159).

#### 3.3.5 Clinical translation

The therapeutic evidence base is strongest in EGPA. Mepolizumab improved remission and relapse outcomes versus placebo in the MIRRA trial (23), and benralizumab was noninferior to mepolizumab in the MANDARA trial while producing deeper peripheral eosinophil depletion (24). These trials validate IL-5–axis targeting for selected EGPA populations but do not establish that complete eosinophil depletion is superior, nor do they justify use in GPA/MPA without dedicated trials. Treatment selection should integrate phenotype, organ involvement, glucocorticoid burden, comorbid eosinophilic disease, and long-term safety rather than eosinophil count alone. Real-world cohorts and evidence syntheses also report clinical and glucocorticoid-sparing benefits of anti-IL-5/IL-5R therapies in EGPA (160–162).

### 3.4 Mechanisms of eosinophil participation in the pathogenesis of autoimmune neurological diseases

Autoimmune neurological disorders provide a stringent test of eosinophil causality because access to central nervous system (CNS) tissue is limited and animal models are heavily used. EOS may contribute to selected eosinophil-rich lesions through trafficking across vascular barriers, granule toxicity, and local immune cross talk, but their importance differs markedly among neuromyelitis optica spectrum disorder, multiple sclerosis (MS), and experimental autoimmune encephalomyelitis (EAE). Human pathology and model-specific evidence are therefore considered separately (163–169). Evidence linking type 2 immune pathways to neurodegenerative disease provides broader neuroimmune context but does not establish eosinophil causality in autoimmune CNS disease (170).

#### 3.4.1 Core universal mechanisms of eosinophil-mediated CNS autoimmune injury

The pathological process of eosinophil involvement in autoimmune neurological diseases relies on a complete cascade of “peripheral activation–blood–brain barrier penetration–central infiltration–degranulation injury–immune microenvironment remodelling.” This pathway differs from its mode of action in peripheral allergic diseases, possessing CNS microenvironment specificity, and can form a positive feedback loop with adaptive immune dysregulation, continuously driving the progression of neuroinflammation.

- Peripheral pre-activation and chemotactic enrichment: peripheral immune imbalance-mediated pre-activation and chemotactic enrichment of EOS are prerequisites for the initiation of neuroinflammation. Patients with autoimmune neurological diseases exhibit significant abnormalities in type 2 immune responses, with sustained high expression of typical type 2 cytokines such as IL-4, IL-5, and IL-13. Among these, IL-5 is the core cytokine driving the proliferation, maturation, and peripheral activation of bone marrow EOS, significantly enhancing their chemotactic sensitivity and degranulation potential (165). Simultaneously, CNS inflammatory lesions and peripheral immune organs can specifically secrete eosinophil chemokines such as CCL11 (Eotaxin-1) and CCL26. By binding to the highly expressed CCR3 receptor on the eosinophil surface, these form a precise chemotactic gradient, inducing peripherally activated EOS to penetrate the damaged blood–brain barrier (BBB) and infiltrate central lesions (169). It is noteworthy that previous research generally considered CCL11 to only mediate allergic inflammation. However, the latest mechanistic studies confirm that tumor necrosis factor-α (TNF-α) in the neuroinflammatory microenvironment can significantly upregulate CCL11 expression in fibroblasts and glial cells through the p38 MAPK-NF-κB signaling pathway, forming a positive feedback loop of “inflammation–chemotaxis–infiltration,” continuously recruiting EOS to accumulate in CNS injury regions (171). More groundbreaking research has confirmed that CCL11 can exacerbate neuronal excitotoxicity and death by activating microglia to produce reactive oxygen species (ROS), establishing a direct link between eosinophil chemotactic signals and irreversible neuronal damage (172).
- Degranulation-mediated toxic injury: centrally infiltrated EOS mediate multi-level neural tissue injury through degranulation and release of toxic proteins, which is their core pathogenic effect. Activated EOS can release specific toxic granule proteins such as eosinophil cationic protein (ECP), eosinophil peroxidase (EPO), and MBP, among which ECP is a key effector molecule in central neuroinflammation (173). Clinical studies have confirmed that ECP levels in the cerebrospinal fluid of some MS patients are significantly elevated and positively correlated with the degree of neurological deficit and lesion activity, suggesting that eosinophil granule proteins can serve as potential biomarkers of central inflammatory activity (173). In the CNS microenvironment, these toxic proteins can directly damage oligodendrocytes, disrupt myelin sheath structure, induce neuronal apoptosis, and simultaneously injure vascular endothelial cells, further exacerbating the increase in blood–brain barrier permeability and creating conditions for more immune cell infiltration (174). Unlike acute oxidative damage from neutrophils, eosinophil-mediated neural injury is characterized by persistence, low intensity, and chronic accumulation, representing an important reason for the chronic perpetuation of neuroinflammation and persistent axonal injury.
- Immune microenvironment remodelling: EOS remodel the local immune microenvironment, breaking central immune tolerance and amplifying the autoimmune response. Traditionally, the CNS was considered to be dominated by type 1 immune responses (Th1/Th17) mediating demyelinating inflammation, while type 2 immune responses were thought to have an immunoprotective role (175). However, recent research has overturned this singular understanding, supporting the interpretation that infiltrating EOS can bidirectionally regulate the differentiation of central T-cell subsets by secreting cytokines, chemokines, and co-stimulatory molecules. They can promote Th2 responses via IL-4/IL-13 while also activating Th17 cells through antigen-presenting functions, exacerbating autoimmune injury (164). Simultaneously, EOS can activate microglia, promote central inflammatory cytokine storms, and inhibit the immunosuppressive function of regulatory T cells (Tregs), completely disrupting CNS immune homeostasis and forming a chronic autoimmune inflammatory loop (165). It is critically worth noting that most current mechanistic research is based on animal models. The complexity of the human CNS microenvironment far exceeds that of model systems. Whether the immunoregulatory function of EOS exhibits cell concentration-dependent and spatiotemporally specific functional conversion in the human body has not been fully elucidated, representing a limitation of current mechanistic research.

#### 3.4.2 Specific pathological roles of eosinophils in classical autoimmune neurological diseases

The degree of infiltration, activation status, and functional effects of EOS exhibit significant disease specificity. They are core immune markers distinguishing clinically similar but pathologically distinct conditions like neuromyelitis optica spectrum disorder (NMOSD) and MS and are key mechanisms explaining the differences in treatment responses and prognostic stratification between these diseases.

##### 3.4.2.1 Neuromyelitis optica spectrum disorder: specific pathogenic injury mediated by eosinophils

NMOSD is a specific autoimmune CNS disease targeting aquaporin-4 (AQP4). Previously long classified as a subtype of MS, the differences in pathological and immune characteristics are the core basis for their differentiation, with significant eosinophil infiltration and activation being a hallmark pathological feature distinguishing NMOSD from MS (167, 168, 176). A large body of clinical pathological studies has confirmed that acute-phase lesions in the spinal cord, optic nerve, and brainstem of NMOSD contain massive eosinophil infiltration, accompanied by significant perivascular inflammatory exudation, complement activation, and tissue necrosis. In contrast, eosinophil infiltration is extremely rare in MS lesions, with only very few severe cases showing sporadic eosinophil distribution (177). This pathological difference directly determines the inflammatory intensity and injury pattern of the two diseases: NMOSD is dominated by acute necrotizing inflammation with severe axonal injury and tissue cavitation, whereas MS is characterized mainly by chronic demyelination and mild inflammatory infiltration (177).

At the molecular mechanism level, EOS and AQP4-IgG–mediated humoral immune dysregulation form a potent synergistic pathogenic effect, constituting the core pathogenic pathway of NMOSD. After AQP4-IgG binds to the AQP4 antigen on the surface of astrocytes, it activates the classical complement pathway, inducing astrocyte injury and death. This, in turn, releases a large amount of chemokines and inflammatory mediators, specifically recruiting peripheral EOS to infiltrate the lesion site (178). Infiltrated EOS can further amplify astrocyte injury and exacerbate AQP4 protein loss through dual mechanisms of antibody-dependent cell-mediated cytotoxicity (ADCC) and complement-dependent cytotoxicity (CDCC), forming a vicious cycle of “autoantibody injury–eosinophil infiltration–tissue necrosis” (174). Clinical studies have reported elevated eosinophil-related chemokines in acute NMOSD and during remission, although the measured analytes and sampling compartments differ between studies (171, 179). These findings suggest involvement of eosinophil recruitment pathways but do not establish continuous elevation throughout the disease course or a causal relationship with relapse and disability.. It is noteworthy that clinical findings show some AQP4-IgG-positive NMOSD patients can present with acute eosinophilic meningoencephalitis, characterized by a significant increase in peripheral blood and cerebrospinal fluid EOS and widespread central inflammatory necrosis, further supporting the interpretation that EOS are key effector cells in the severe phenotype of NMOSD (180).

A critical analysis of existing research reveals a significant cognitive bias in the current NMOSD field: most studies focus on the role of AQP4-IgG and complement, viewing eosinophil infiltration as a secondary inflammatory manifestation, thereby neglecting their active pathogenic function. In fact, clinical intervention studies targeting EOS have shown that antihistamines and eosinophil inhibitors can significantly reduce the relapse rate of NMOSD and alleviate the degree of lesion inflammation, supporting the interpretation that EOS are not passive companions but targets actively participating in disease progression (181). Furthermore, the persistently elevated eosinophil chemotactic factors in NMOSD patients during remission suggest they may be involved in relapse warning and the maintenance of immune homeostasis imbalance. However, the specific regulatory mechanisms remain unclear and represent an important direction for future precision diagnosis and treatment research (182).

##### 3.4.2.2 Multiple sclerosis and experimental autoimmune encephalomyelitis: the controversy over the bidirectional function of eosinophils

MS, as the most common chronic inflammatory demyelinating disease of the CNS, is pathologically characterized predominantly by CD4^+^ T cell–mediated type 1 immune responses, diffuse demyelination, and lymphocyte infiltration. Previous research almost completely negated a pathological role for EOS (183). However, recent refined pathological analyses and animal model studies have completely rewritten this understanding, supporting the interpretation that EOS exert a significant spatiotemporally dependent bidirectional function in MS and the classic animal model EAE. That is, they play pathogenic damaging roles in certain disease stages and immune microenvironments, and protective immunomodulatory roles in others. This is the core reason for the long-standing research controversy in this field (168).

- Pathogenic aspect: in a minority of severe MS, acute fulminant MS, and drug-induced MS neural injury, EOS play a key inflammation-amplifying role. Clinical cases show that some MS patients, after receiving disease-modifying therapies like natalizumab, daclizumab, or dimethyl fumarate, can develop eosinophilia, eosinophilic fasciitis, or even drug reaction with eosinophilia and systemic symptoms (DRESS) syndrome, accompanied by acute exacerbation of central neuroinflammation and worsening neurological deficits. This confirms that eosinophil activation can directly mediate MS-related neural injury (184–186). Mechanistic studies indicate that low-level expression of CCL11 in MS lesions can recruit a small number of EOS to infiltrate, releasing toxic granule proteins that exacerbate local demyelination and axonal injury. Leukotriene B4 (LTB4) can directly influence the severity of EAE by regulating the efficiency of eosinophil infiltration, blocking the LTB4 receptor significantly inhibits eosinophil infiltration and reduces central inflammation (187). Meanwhile, the ECP positivity rate in the cerebrospinal fluid of MS patients can reach 25%, suggesting that eosinophil activation is a potential pathological feature of a subset of MS patients and can be used to explain the heterogeneity in clinical phenotypes and prognoses (173).
- Protective aspect: multiple EAE model studies have confirmed that type 2 immune responses and eosinophil activation can significantly suppress central autoimmune inflammation and alleviate clinical disease symptoms. Parasite-secreted products can inhibit central inflammatory infiltration and demyelinating injury in the EAE model and improve neurological function by inducing IL-5– and IL-33–mediated eosinophilia (188). The mechanism is that appropriately activated EOS can remodel the CNS immune tolerance microenvironment by inhibiting Th1/Th17 cell differentiation, promoting Treg functional activation, and downregulating levels of central inflammatory factors, thereby exerting an immunoprotective effect (175). The core regulatory mechanism of this bidirectional function is closely related to the degree of eosinophil activation, cell density, disease course, and the cytokine profile of the microenvironment. In the acute phase, high concentrations of excessively activated EOS exert pathogenic damaging effects, while in the chronic phase, low concentrations of homeostatically activated EOS exert immunomodulatory effects (165).

From a critical perspective, the limitations of eosinophil research in the MS field lie in the disconnect between clinical and basic research and the neglect of population heterogeneity. Existing animal model studies mostly focus on systemic eosinophil regulation and cannot mimic the spatiotemporal distribution characteristics of EOS in focal MS lesions in humans. Simultaneously, MS patients exhibit significant heterogeneity in subtypes, disease courses, and treatment regimens. The activation status and functional effects of EOS differ significantly among different patient subgroups, which is also the core reason for the inconsistency in conclusions from multiple clinical studies. Future research needs to dissect the precise functional mechanisms of EOS in MS based on subgroup stratification.

#### 3.4.3 Eosinophil abnormalities and neurotoxicity related to targeted therapy in autoimmune neurological diseases

Clinical targeted drugs for autoimmune neurological diseases can induce aberrant activation and infiltration of EOS in the periphery and CNS by modulating systemic immune homeostasis, leading to secondary neural injury and systemic adverse reactions. This “treatment-related eosinophil toxicity” is a pathological phenomenon easily overlooked in clinical practice and an important piece of clinical evidence for eosinophil involvement in neuroimmune diseases (184). It has been clearly established that the first-line MS drugs natalizumab, daclizumab, and dimethyl fumarate are all associated with eosinophil-related adverse reactions.

Natalizumab, as a leukocyte chemotaxis inhibitor, treats relapsing-remitting MS by blocking leukocyte migration across the blood–brain barrier. However, clinical studies have found that this drug can induce peripheral blood eosinophilia, pulmonary eosinophilia, and eosinophilic fasciitis. Some patients may experience worsening central neuroinflammation. The mechanism is drug-mediated systemic immune imbalance, inducing excessive activation of type 2 immune responses, promoting eosinophil proliferation and activation, and subsequently inducing multi-organ eosinophilic inflammatory damage (185, 189). Daclizumab treatment for MS can induce severe DRESS syndrome, characterized by significant eosinophilia, systemic inflammatory response, and acute neurological deterioration, which can be life-threatening in severe cases, directly supporting the interpretation that excessive eosinophil activation can mediate fatal neuroimmune injury (184, 190). Although dimethyl fumarate has a good safety profile, it can indirectly regulate eosinophil infiltration by inhibiting CCL11 expression through the p38 MAPK-NF-κB pathway. Some patients may experience eosinophil-related skin and fascial inflammation after medication, suggesting its regulatory effect on the eosinophil pathway is bidirectional (171, 186).

Furthermore, B cell–depleting agents like rituximab, commonly used for NMOSD and MS, primarily target B cells. However, clinical cases have found that they can induce tuberculosis infection and systemic immune dysregulation, indirectly affecting eosinophil homeostasis and leading to secondary fluctuations in neuroinflammation (191). These clinical phenomena fully confirm that eosinophil homeostatic imbalance is an important factor influencing the treatment prognosis of autoimmune neurological diseases. Precisely regulating the level of eosinophil activation is a key breakthrough point for optimizing existing treatment regimens and reducing drug adverse reactions.

#### 3.4.4 Evidence limitations in autoimmune neurological disease

Human evidence is strongest where EOS are directly observed in lesions or cerebrospinal compartments, whereas much of the mechanistic literature relies on EAE. EAE is useful for pathway testing but does not recapitulate the full cellular or molecular biology of human MS, and murine eosinophil markers cannot be assumed to identify equivalent human states. Proposed neuroprotective and neurotoxic eosinophil functions should therefore be labelled by species and disease model.

### 3.5 Mechanisms of eosinophil participation in the pathogenesis of autoimmune endocrine diseases

In autoimmune endocrinopathies, evidence for eosinophil involvement is substantially weaker than in EGPA or eosinophil-rich skin disease. Studies in type 1 diabetes and Graves’ disease report peripheral, genetic, transcriptomic, or experimental signals, but direct demonstration of an eosinophil-dependent tissue mechanism is limited. The following sections therefore treat EOS as candidate modulators within broader immune networks rather than established disease-driving cells (189–193).

#### 3.5.1 Bidirectional immunomodulation and pathogenic mechanisms of eosinophils in type 1 diabetes

T1DM is the most representative autoimmune endocrine disease, with the core pathological feature being the specific infiltration of autoreactive T cells into the pancreatic islets, progressively destroying insulin-secreting β cells and ultimately leading to absolute insulin deficiency (194). The traditional T1DM pathogenesis model was dominated by Th1-type immune responses. However, recent research has confirmed that type 2 immune activation and abnormal EOS infiltration and functional reprogramming are key intermediate links connecting immune dysregulation and islet injury, breaking the singular perception that “T1DM is merely a Th1-dominant disease” (195). The role of EOS in T1DM exhibits significant spatiotemporal bidirectionality: the pre-disease phase is dominated by immune regulation and initiation of tolerance breakdown, the active progression phase is centered on direct tissue injury and amplification of inflammatory cascades, and the end-stage is involved in local fibrosis and complication progression, forming a multi-stage, multidimensional pathogenic network (194, 196).

Genome-wide association studies provide underlying genetic evidence for EOS involvement in T1DM pathogenesis, overcoming the limitation of previous research that solely focused on immune function phenotypes. Genome-wide genetic correlation analysis confirms a significant positive genomic genetic association between T1DM and peripheral blood EOS count [*r*(g) = 0.093, *P* < 0.05], suggesting that they share core genetic susceptibility loci and that EOS count abnormality is a genetic risk marker for T1DM rather than simply a secondary change due to inflammation (197). Bidirectional Mendelian randomization studies further supplement that an abnormal EOS proportion is a shared risk factor for the occurrence of T1DM and can independently participate in the comorbidity pathogenesis of T1DM and diabetic nephropathy, clarifying the causal regulatory trend of EOS dysregulation on T1DM and overturning the one-sided conclusions of some earlier studies that “peripheral blood EOS count is not statistically associated with T1DM onset” (198, 199). This coupling at the genetic level fundamentally explains the immunological basis of the widespread EOS functional abnormalities in T1DM patients and also suggests that EOS-related genetic loci can serve as novel genetic markers for screening high-risk populations for T1DM.

At the cellular and molecular mechanism level, specifically activated functional EOS subsets exist in T1DM patients, whose phenotype is significantly different from normal physiological EOS. Classical studies, through cell surface marker identification, specific gene expression detection, and confocal microscopy validation, have clarified that EOS in the peripheral blood and pancreatic infiltrates of T1DM patients exhibit a specific CD15/CD14 phenotype. This subset is in a state of sustained transcriptional activation, highly expressing toxic effector molecules such as myeloid α-defensins and myeloperoxidase, possessing the ability to directly mediate tissue oxidative damage and amplify local inflammation (200). High-dimensional CyTOF immune profiling analysis further confirms that long-term T1DM patients have a large number of infiltrating EOS detectable in pancreatic tissue, and that EOS can specifically regulate T-cell function by secreting galectin-10, achieving targeted regulation of adaptive immune responses. This is also the core molecular pathway through which EOS connect innate and adaptive immunity and break islet immune tolerance (194). Unlike traditional immunosuppressive effects, the T-cell regulation mediated by EOS in the T1DM microenvironment is bidirectional: under physiological conditions, galectin-10 can maintain peripheral immune tolerance, but under sustained inflammatory stimulation, overactivation of EOS leads to an imbalance in this regulatory pathway, inhibiting regulatory T-cell (Treg) function and activating autoreactive effector T cells, thereby accelerating specific immune-mediated damage to islet β cells (194, 201).

Animal model studies have precisely recreated the dynamic pathological process of EOS involvement in T1DM pathogenesis, filling the mechanistic validation gap that human clinical studies cannot achieve due to temporal constraints. Research on the BioBreeding (BB) spontaneously diabetic rat model (mimicking human spontaneous T1DM) shows that diabetes-prone DRlyp/lyp rats exhibit abnormally high expression of the eosinophil chemokine eotaxin in the pancreas in the pre-diabetic phase, which can specifically recruit circulating EOS to infiltrate islet tissue, forming early insulitis pathological changes, a feature not seen in non-susceptible rats (202). Infiltrated EOS can directly induce β-cell apoptosis by releasing granular toxic proteins and pro-inflammatory cytokines, synergizing with mast cells and macrophages to amplify local islet inflammation (203). The streptozotocin-induced T1DM-like mouse model further confirmed that over 80% of the circulating Ly6G^+^CD49d^+^ pro-angiogenic granulocyte subset are EOS. This EOS subset highly expresses CXCR4 and VEGFR1, can specifically infiltrate pancreatic tissue, remodel the local islet microenvironment, and exacerbate metabolic disorders and immune injury (204). Notably, antioxidant intervention studies have found that N-acetylcysteine can alleviate the severity of insulitis and significantly delay the onset of T1DM by regulating peripheral EOS abundance and inhibiting EOS-mediated oxidative inflammatory injury. However, it cannot completely block disease occurrence, suggesting that EOS are an important regulatory factor in T1DM pathogenesis but not the sole determinant factor, precisely defining their weight within the pathogenic network (196).

In clinical scenarios, EOS abnormalities not only participate in the primary pathogenesis of T1DM but also mediate treatment-related adverse reactions and complication progression, forming a complete disease regulatory chain. In immune checkpoint inhibitor-induced secondary T1DM (ICI-T1DM), a rare severe autoimmune endocrine injury, the peripheral blood neutrophil/eosinophil ratio is significantly elevated. This ratio imbalance can serve as a novel inflammatory marker for early warning of ICI-T1DM, reflecting the degree of systemic immune dysregulation (205). In the setting of insulin replacement therapy, the skin tissue at the injection sites of patients on continuous subcutaneous insulin infusion can exhibit significant EOS infiltration, accompanied by local fibrosis, fat necrosis, and chronic inflammation, suggesting that EOS-mediated local allergy-like inflammatory responses are a core trigger for local complications of insulin therapy (206, 207). Furthermore, the leukotriene pathway is a key regulatory pathway for EOS activation and degranulation. Its inhibitor, montelukast, can alleviate T1DM-related inflammatory injury by inhibiting EOS activation and reducing the release of toxic granules, offering a new target for T1DM adjuvant therapy and inversely validating the pathogenic function of EOS (208).

Critically addressing current research controversies, it must be pointed out that some cross-sectional clinical studies showing no significant abnormality or even a mild decrease in peripheral blood EOS counts in newly diagnosed T1DM patients do not contradict genetic and histopathological research findings. Instead, they reveal the spatiotemporal and tissue specificity of EOS action (209). Peripheral blood EOS counts cannot fully reflect local pancreatic EOS infiltration and activation status. In the early disease stage, EOS can massively chemotactically migrate to pancreatic lesions, leading to a transient decrease in peripheral blood. As the disease progresses and systemic immune dysregulation intensifies, peripheral blood EOS functional abnormalities and ratio imbalances gradually become apparent (198, 199). This also explains why single peripheral blood cell counts cannot serve as diagnostic markers for T1DM, whereas EOS functional status, genetic susceptibility, and tissue infiltration characteristics better reflect their pathogenic value.

#### 3.5.2 Immune dysregulation mechanisms and clinical value of eosinophils in Graves’ disease

GD is the most common organ-specific autoimmune thyroid disease. Its core pathogenesis involves the aberrant activation of thyroid follicular cells by thyrotropin receptor antibodies (TRAb), leading to hyperthyroidism, accompanied by Th2-dominant immune dysregulation, local chronic inflammation, and tissue remodelling (193). Previous research on GD immune mechanisms focused on B cell–mediated autoantibody secretion and T-cell aberrant activation. Recent studies have clearly identified EOS as key effector cells in the type 2 immune imbalance of GD, participating in the entire process of disease occurrence, progression, relapse, and extra-thyroidal complications (Graves’ ophthalmopathy). Moreover, their peripheral blood and histological features can accurately differentiate GD from subacute thyroiditis, holding significant clinical translational value (204, 210).

Clinical cohort studies have clarified the existence of a specific abnormal EOS phenotype in GD patients, closely related to disease activity and immune subtypes. Large-sample controlled studies confirm that the proportion of peripheral blood EOS in untreated, newly diagnosed GD patients is significantly higher than in healthy individuals and patients with subacute thyroiditis. This feature can serve as a core differential indicator for the two types of hyperthyroidism: subacute thyroiditis is dominated by Th1-type inflammation with significantly reduced peripheral blood EOS, while GD is characterized by type 2 immune activation and EOS recruitment and activation, providing a novel biological basis for non-invasive differential diagnosis (210). Further subtyping studies show that GD patients in the high IgG4/IgG ratio subgroup have significantly elevated peripheral blood EOS counts, IL-1β, and IL-17 levels, suggesting that EOS activation is closely related to the IgG4-related immune subtype of GD and participates in the immunopathological process of severe, refractory GD (22). Meanwhile, serum levels of eosinophil-derived neurotoxin (EDN) are significantly elevated in GD patients, and the expression level of EDN is positively correlated with disease activity and thyroid autoantibody titers, supporting the interpretation that toxic mediators released by EOS degranulation can serve as novel serum markers of GD disease activity (211).

EOS mediate the occurrence and relapse of GD by regulating the Th1/Th2 immune balance and amplifying autoimmune inflammation, with cross-regulatory mechanisms existing between allergic inflammation and GD. Epidemiological and clinical intervention studies have confirmed that after the onset of Th2-type inflammatory diseases like allergic rhinitis, hyperthyroid symptoms in GD patients are prone to relapse and autoantibody titers increase significantly. The mechanism is that allergic inflammation induces massive systemic activation and proliferation of EOS, releasing type 2–associated cytokines such as IL-4 and IL-13. This further amplifies the Th2 immune dominance in GD patients, promotes B-cell proliferation and differentiation, and aberrant secretion of TRAb, forming a vicious cycle of “allergic inflammation–EOS activation–GD relapse” (212, 213). This mechanism perfectly explains the comorbidity characteristics of GD and allergic diseases and clearly identifies EOS as a key bridging cell connecting allergic inflammation and thyroid autoimmune dysregulation. Compared with healthy individuals, EOS in GD patients are more easily activated by inflammatory stimuli and have a stronger degranulation capacity, which can sustain and amplify local chronic thyroid inflammation, induce thyroid follicular cell proliferation and hyperfunction, and maintain the chronic active state of the disease (202).

In extra-thyroidal complications, EOS specifically participate in the pathological processes of Graves’ ophthalmopathy and local thyroid fibrotic remodelling. As the most common refractory complication of GD, Graves’ ophthalmopathy patients exhibit significantly elevated EOS-related inflammatory markers in peripheral blood, and the degree of EOS infiltration is highly correlated with the severity of extraocular muscle inflammation and fibrosis (199). In thyroid autoimmune fibrotic diseases such as invasive fibrous thyroiditis, thyroid tissue shows massive EOS infiltration and extensive degranulation. The released MBP can directly induce fibroblast proliferation and collagen deposition, mediating glandular fibrotic remodelling. In contrast, normal thyroid tissue and Hashimoto’s thyroiditis tissue only show a small number of quiescent EOS without significant degranulation, suggesting that EOS-mediated fibrotic injury is the mechanism of long-term structural destruction in thyroid autoimmune diseases (214). Moreover, adverse reactions related to antithyroid drug therapy are also closely associated with aberrant EOS activation. Propylthiouracil and methimazole treatment can induce EOS-mediated autoimmune drug injuries such as eosinophilic pleurisy and vasculitis, further confirming the core effector role of EOS in thyroid autoimmune disorders and secondary inflammatory injury (215, 216).

#### 3.5.3 Evidence synthesis for autoimmune endocrinopathies

In type 1 diabetes and Graves’ disease, EOS may reflect broader type 2 or tissue-remodelling programs, but direct causal evidence is limited. Peripheral counts and transcriptomic deconvolution cannot substitute for tissue-resolved functional data. We therefore interpret endocrine associations as hypothesis-generating and do not infer a disease-driving eosinophil subtype or a therapeutic indication for eosinophil depletion.

### 3.6 Mechanisms of eosinophil participation in the pathogenesis of autoimmune skin diseases

Autoimmune and immune-mediated skin diseases span conditions in which EOS are major tissue effectors and others in which they are variable bystanders. Bullous pemphigoid provides strong pathological evidence for eosinophil recruitment and degranulation, whereas psoriasis and connective-tissue-disease skin lesions show greater heterogeneity. This section emphasizes disease-specific histology and mechanism rather than using “skin eosinophilia” as a single biological category (202–207).

#### 3.6.1 Psoriasis: variable tissue eosinophilia without an established severity biomarker

EOS are not a defining histological feature of psoriasis, but they are detectable in a subset of lesions. A 2024 systematic review and meta-analysis of five diagnostic-pathology studies (218 pooled cases) estimated dermal EOS in 46% of specimens (95% CI 27%–66%), with substantial between-study heterogeneity (*I*² = 89%) (22). These data support heterogeneity in eosinophil presence but do not establish a consistent correlation with psoriasis severity or a causal role. Eosinophil-rich lesions should therefore prompt consideration of disease variant, treatment effects, and differential diagnoses rather than being interpreted as a universal psoriasis endotype.

From the perspective of chemotaxis and activation mechanisms, skin barrier damage and microbial dysbiosis constitute upstream initiating signals for eosinophil recruitment. Staphylococcus aureus colonizing the damaged epidermis of psoriasis can secrete various enterotoxins and virulence factors, activating keratinocytes and dendritic cells to release a large amount of chemotactic mediators, synergistically recruiting circulating EOS to migrate into the dermis (217); Malassezia can also indirectly amplify the local inflammatory microenvironment by activating the keratinocyte IL-23 pathway, promoting myeloid cell activation (218). The epithelium-derived alarmin IL-33/ST2 axis is a key pathway connecting skin damage and eosinophil activation. Keratinocyte necrosis and barrier disruption lead to the release of IL-33, which binds to the ST2 receptor on the eosinophil surface, inducing eosinophil degranulation. This releases eosinophil cationic protein (ECP), MBP, and galectin-10 (Charcot-Leyden crystal protein), directly damaging the keratinocyte cell membrane, further exacerbating epidermal spongiosis and compromising barrier integrity, forming a positive feedback loop of “barrier injury–alarmin release–eosinophil activation–further barrier disruption” (122, 219). Animal experiments further support a trans-organ effect: in the imiquimod-induced psoriasis-like mouse model, TLR7-dependent eosinophil degranulation not only drives skin inflammation but also induces intestinal barrier damage, establishing a skin-gut immune axis interaction (220).

The most critical controversy in the current field centres on the determination of causality. Mendelian randomization studies show that genetically predicted elevated peripheral blood EOS are significantly associated with the risk of atopic dermatitis, but the causal association evidence with psoriasis onset is currently insufficient (221). Two mutually exclusive hypotheses coexist: the first hypothesis posits that eosinophil infiltration is a secondary accompanying event under sustained chronic inflammatory stimulation, passively recruited by a large number of pro-inflammatory factors within the lesion, only amplifying local tissue injury and not participating in disease initiation; the second hypothesis proposes the existence of a unique psoriasis endotype, namely, “eosinophil-rich psoriasis,” where EOS actively regulate the functions of dendritic cells and macrophages, modulate Th17 cell differentiation, and remodel the local immune microenvironment. Clinically observed eczematous psoriasis is the most typical representative of this endotype, accounting for approximately 5%–10% of all psoriasis patients, simultaneously possessing dual characteristics of Th17 and type 2 inflammation, easily confused with atopic dermatitis, and highlighting the limitations of relying solely on Th17 pathway targeting (222).

Peripheral biomarker studies provide further clues for stratification (223). Multiple retrospective cohorts have confirmed that the baseline eosinophil/lymphocyte ratio (ELR) can predict the treatment response to IL-23 inhibitors like guselkumab: patients with a lower baseline ELR are more likely to achieve long-term PASI90/PASI100 responses, suggesting that peripheral eosinophil levels can serve as potential biomarkers for personalized treatment stratification in psoriasis (224, 225). However, the existing bottleneck lies in the inability to distinguish between the phenotypes of circulating EOS and tissue-infiltrating EOS; there is a lack of targeted interventions that can specifically block skin eosinophil infiltration without affecting systemic immune homeostasis, making it impossible to conduct reliable causal validation intervention trials. In addition, paradoxical psoriatic lesions induced by immune checkpoint inhibitors also exhibit significant eosinophil infiltration, suggesting that under conditions of adaptive immune imbalance, EOS can be activated by various immune perturbation signals and participate in the process of autoimmune skin injury (226).

#### 3.6.2 Core effector functions of eosinophils in autoimmune blistering skin diseases

Autoimmune blistering diseases are a classic spectrum of autoimmune skin disorders mediated by autoantibodies against skin structural proteins, encompassing pemphigoid and pemphigus spectrums. Previous mechanistic research centered on complement activation and neutrophil recruitment following autoantibody binding to target antigens. In recent years, increasing evidence has confirmed that EOS are key effector cells determining the severity of blister formation and tissue erosion, playing an irreplaceable role, especially in bullous pemphigoid (BP), pemphigus herpetiformis, and pemphigus vegetans subgroups.

Bullous pemphigoid, as the most prevalent autoimmune blistering disease in the elderly, possesses two characteristic immunological phenotypes: circulating anti-BP180/BP230 IgG autoantibodies, and significant peripheral blood and lesional eosinophilia (227). Clinical data show that approximately 80% of patients with active BP have elevated peripheral blood EOS, and the eosinophil count is positively correlated with the number of blisters and disease activity. Histopathologically, it is characterized by a large number of EOS infiltrating the superficial dermis and subepidermal blistering. In early lesions, EOS can be observed migrating into the epidermis, forming eosinophilic spongiosis. Mechanistically, the binding of anti-BP180 antibodies to basement membrane zone antigens activates the complement system, producing potent chemokines like C5a, which rapidly recruit circulating EOS to migrate to the dermal-epidermal junction. Activated EOS release granule proteins, directly inducing collagen degradation in the basement membrane zone and basal-side apoptosis of keratinocytes, promoting subepidermal blister formation. Concurrently, activated EOS secrete type 2 cytokines such as IL-4 and IL-13, further promoting B-cell proliferation and sustained autoantibody production, forming a vicious cycle of “autoantibody–complement activation–eosinophil activation–more tissue damage and antibody generation.” Notably, dupilumab (anti–IL-4Rα) can significantly reduce peripheral blood EOS and control skin lesions in refractory BP cohorts, clinically confirming the important role of the type 2 cytokine–eosinophil axis in BP pathogenesis (228).

The role of EOS in the pemphigus spectrum of diseases exhibits significant subtype heterogeneity. Typical lesions of pemphigus vulgaris and pemphigus foliaceus are dominated by acantholysis and neutrophil infiltration, with sparse EOS. However, pemphigus herpetiformis and Hallopeau-type pemphigus vegetans almost invariably present with characteristic eosinophilic spongiosis, with a large number of eosinophils infiltrating within the epidermis, and Charcot-Leyden crystals detectable in the tissue (49, 229). Pemphigus herpetiformis combines the clinical manifestation of annular blisters with severe pruritus and the immunofluorescence features of pemphigus, long posing a diagnostic dilemma. Eosinophil enrichment within the skin lesions is an important pathological distinguishing feature. In early lesions, intraepidermal eosinophil infiltration can be preferentially observed before acantholysis appears, giving rise to a key scientific conjecture: eosinophil activation may participate in the early disruption of keratinocyte adhesion function, rather than merely being a secondary inflammatory infiltration following acantholysis (230). In contrast, mucosal lesions in most cases of pemphigus vulgaris are dominated by lymphocyte infiltration, with sparse EOS, suggesting that the distribution of target antigens and the local cytokine microenvironment jointly determine the recruitment efficiency of EOS.

A prominent open question in the current field is why, given the presence of autoantibodies against desmosomal proteins, does the degree of eosinophil infiltration vary so drastically among different pemphigus subgroups? Existing hypotheses point to the regulatory roles of autoantibody subtypes, antigenic epitope differences, and the expression levels of local skin microbiota and alarmins. Furthermore, it is unknown whether EETs participate in tissue injury in blistering diseases. It has been confirmed in other skin inflammation models that activated EOS can release DNA mesh-like structures called EETs, amplifying local inflammation and continuously activating innate immunity. However, systematic research on the expression, function, and clinical relevance of EETs in autoimmune blistering diseases is still lacking (231, 232).

#### 3.6.3 Eosinophils in connective tissue disease-associated autoimmune skin lesions: overlooked heterogeneous participants

Skin lesions associated with connective tissue diseases such as dermatomyositis, scleroderma, and lichen sclerosus have long been classified under the category of “traditional eosinophil-poor inflammatory skin diseases.” A pathological study aggregating various rare inflammatory skin diseases has confirmed that varying numbers of infiltrating EOS can be detected in biopsy specimens of dermatomyositis and lichen sclerosus. Moreover, skin lesions with eosinophil enrichment tend to be accompanied by more pronounced skin sclerosis, pruritus, and a chronic disease course (233).

The characteristic skin manifestations of dermatomyositis include heliotrope rash, Gottron’s papules, and poikiloderma, with the core pathology being interface dermatitis, where infiltrating cells are predominantly CD4^+^T and CD8^+^T lymphocytes. However, an increasing number of case cohorts have found that some dermatomyositis patients’ skin lesions are accompanied by dermal eosinophil infiltration and peripheral blood eosinophilia. These patients are more likely to develop refractory skin lesions and respond poorly to conventional glucocorticoids and immunosuppressants. Some subgroups, such as antisynthetase syndrome-associated dermatomyositis and amyopathic dermatomyositis, can exhibit upregulated levels of eotaxin, a chemoattractant for EOS, suggesting that innate immune signals drive the local recruitment of EOS (234). Similarly, in the acral sclerosis of systemic sclerosis and lichen sclerosus lesions, TGF-β and granule proteins released by EOS participate in fibroblast activation and excessive collagen deposition, transforming immune inflammatory signals into a tissue fibrosis phenotype, thus building a bridge for the transition from inflammation to fibrosis. This mechanism also suggests that EOS are not limited to acute inflammatory injury but also participate in the later-stage tissue remodelling processes of chronic autoimmune skin diseases.

Beyond the diseases mentioned above, chronic spontaneous urticaria, as an inflammatory skin disease with autoimmune features, has functional autoantibodies against IgE receptors in about half of the patients. Mast cell degranulation is the core initiating event, but EOS, as key synergistic cells, continuously amplify the increase in vascular permeability and the pruritus response. EOS form a complex cellular interaction network with mast cells and T cells, maintaining the chronic, recurrent wheal damage to the skin (235). This spectrum of diseases further expands the boundaries, demonstrating that EOS broadly participate in both antibody-mediated and cell-mediated patterns of autoimmune skin injury.

#### 3.6.4 Cross-disease interpretation and translational limits in autoimmune skin disease

The evidentiary gradient across autoimmune dermatoses is steep. In bullous pemphigoid and other eosinophil-rich blistering disorders, eosinophil recruitment and degranulation are closely linked to tissue pathology. In psoriasis and connective-tissue-disease skin lesions, EOS are more variable and may be secondary or phenotype specific. Biomarkers such as ECP, galectin-10/CLC protein, and tissue eosinophil density therefore require disease-specific validation before they can be used for treatment selection.

### 3.7 Mechanisms of eosinophil participation in the pathogenesis of inflammatory bowel disease

IBD comprises ulcerative colitis and Crohn’s disease, both characterized by barrier disruption, microbial dysbiosis, and dysregulated mucosal immunity. EOS are abundant physiological residents of the gastrointestinal mucosa, creating a distinctive interpretive problem: increased density or activation may reflect inflammation, host defence, repair, or fibrosis depending on compartment and disease stage. Accordingly, the relevant question is not whether EOS are present, but which state they occupy and whether that state is mechanistically necessary for pathology (236–240).

#### 3.7.1 Recruitment, activation, and subset heterogeneity of eosinophils in the IBD intestinal microenvironment

Under homeostatic conditions, EOS mainly reside in the lamina propria of the gastrointestinal tract, constituting an innate immune component of the intestinal mucosa, participating in microbiota surveillance and epithelial homeostasis maintenance (241, 242). However, there is a clear transcriptomic and functional boundary between homeostatic eosinophils and inflammation-activated EOS (239). During the pathogenesis of IBD, multiple epithelium-derived alarmins, chemokines, and cytokines jointly drive the production, peripheral migration, and targeted intestinal tissue infiltration of bone marrow EOS. IL-5 is the most critical cytokine regulating eosinophil survival and proliferation. In the inflamed IBD mucosa, ILC2s, activated T cells, and mast cells can continuously secrete IL-5, significantly extending the half-life of circulating EOS. Concurrently, epithelial damage releases TSLP, IL-33, and IL-25, further amplifying type 2 inflammatory signals and synergistically inducing the expression of chemokines such as CCL11 (Eotaxin-1), CCL24, and CCL25, which mediate chemotactic migration through the CCR3 receptor on the eosinophil surface (240). It is worth critically noting that previous studies generally regarded the CCL11-CCR3 pathway as the sole pathway for eosinophil infiltration. However, Mendelian randomization studies suggest that there is no clear unidirectional causal association between circulating eosinophil counts and the risk of developing IBD, indicating that chemotactic infiltration is merely a surface phenomenon and that the local tissue activation status determines the pathological effects of EOS (243, 244).

A landmark single-cell study published in Nature in 2023 by Gurtner et al. confirmed that a population of activated EOS exists in the intestinal mucosa of IBD patients, highly expressing granule proteins and co-stimulatory molecules, closely adjacent to CD4^+^T lymphocytes, and possessing the ability to directly promote T-cell proliferation and cytokine secretion. In contrast, homeostatic intestinal EOS lack co-stimulatory molecule expression and do not have the ability to drive adaptive immune activation (237). This discovery resolves a long-standing controversy: why, despite eosinophil infiltration in the mucosa, some individuals only manifest mild mucosal inflammation while others progress to typical IBD. Recent research further divides intestinal EOS into two major functional subsets: classical pro-inflammatory EOS and tissue-protective EOS. The pro-inflammatory subset rapidly degranulates upon stimulation by oxidative stress and TLR signals; the protective subset continuously expresses COX-2, stabilizing the intestinal epithelial barrier through the PGE_2_-IL-22 pathway and inhibiting the severity of experimental colitis, directly proving that EOS are not a unified pro-inflammatory cell population (238). This subset heterogeneity hypothesis also explains the contradictory results from animal models: systemic depletion of EOS alleviates inflammation in some DSS colitis models but exacerbates intestinal injury under specific conditions.

Beyond the cytokine network, neuro-immune interactions also participate in regulating intestinal eosinophil activity. Neuromedin U (NMU) signals can programmatically regulate the mucosal immune function of small intestinal EOS. In IBD-damaged intestinal segments, neuropeptides released by gut neurons directly regulate eosinophil degranulation, establishing a gut neuro-eosinophil inflammatory axis. This axis participates in functional and organic overlapping gastrointestinal symptoms such as abdominal pain and diarrhea, providing a new perspective for the mechanisms of extra-intestinal symptoms and the brain-gut axis in IBD (245, 246).

#### 3.7.2 Eosinophil degranulation products mediate epithelial barrier disruption and mucosal inflammation amplification

Defects in the intestinal epithelial barrier are one of the core initiating events in IBD. Eosinophil degranulation releases a large amount of cytotoxic granule proteins, constituting an important effector pathway for barrier injury. MBP, eosinophil cationic protein (ECP), eosinophil-derived neurotoxin (EDN), and eosinophil peroxidase (EPO) are the four core toxic mediators (247). MBP can directly disrupt the distribution of tight junction proteins such as occludin and claudin in epithelial cells, increasing intestinal permeability and promoting the translocation of intestinal microbes and their metabolites into the lamina propria, continuously activating mucosal immunity. ECP possesses ribonuclease activity and a cell membrane lytic effect, inducing epithelial cell apoptosis. Clinical cohorts have confirmed that fecal EDN and serum ECP levels are significantly elevated in patients with active IBD, positively correlating with disease activity and endoscopic severity, demonstrating potential as non-invasive biomarkers. Importantly, even when the neutrophil marker fecal calprotectin returns to normal, persistently elevated fecal EDN suggests residual submucosal eosinophilic inflammation, which can predict subsequent clinical relapse (248, 249).

Critically, eosinophil degranulation does not only occur during necrotic cell lysis. Live cell imaging has confirmed that EOS can undergo “selective exocytosis,” locally releasing mediators without complete cell disintegration, enabling sustained, focal chronic injury. This is also an important cytological basis for the multifocal, chronic persistent inflammation characteristic of the IBD mucosa. Compared with the acute explosive inflammation of neutrophils, eosinophil-mediated injury is more insidious and persistent, easily overlooked in histological evaluations—the current IBD histological scoring systems (Geboes score, Nancy score) have long used neutrophil infiltration as the core indicator, generally underestimating eosinophil-mediated subclinical inflammation (250).

#### 3.7.3 The regulatory network centered on reactive oxygen species: an emerging core mechanistic paradigm

Tomii and Kano proposed the eosinophil ROS-centered hypothesis in 2025, reshaping the cognitive framework for the role of EOS in IBD (251). EOS are rich in eosinophil peroxidase and, upon activation, generate large amounts of ROS. ROS exert dual effects: on one hand, they directly induce epithelial oxidative damage and mitochondrial dysfunction, amplifying apoptosis; on the other hand, they act as second messengers to activate multiple inflammatory signaling pathways. ROS can activate NF-κB and the NLRP3 inflammasome, promoting the secretion of IL-1β, TNF-α, and IL-6 by macrophages and dendritic cells, thereby linking type 2 immunity with classical pro-inflammatory pathways. This explains why IBD (traditionally a Th1/Th17 disease) can simultaneously exhibit significant features of type 2 immune activation.

The most important critical value of this hypothesis lies in bridging a long-standing research divide: previous research has treated oxidative stress, eosinophil granule proteins, and the inflammasome as three independent pathways. ROS not only amplify inflammation but also positively promote the sustained activation and degranulation of EOS themselves, forming a self-amplifying inflammatory loop. Simultaneously, ROS can regulate the secretion of chemokines, further recruiting more EOS and neutrophils to aggregate at the lesion site. Based on this mechanism, Siglec-8 has become a highly promising therapeutic target. Siglec-8 is selectively expressed on human EOS. Upon ligand binding, it can inhibit ROS production and induce eosinophil apoptosis, breaking the ROS-mediated positive inflammatory feedback loop and offering a novel intervention approach distinct from the anti-IL-5 pathway for refractory IBD (251).

However, this paradigm still has critical gaps: most of the existing evidence is derived from *in vitro* experiments and rodent colitis models. The spatiotemporal dynamics of eosinophil ROS in the *in-situ* human intestinal microenvironment, and the differences in the degree of ROS pathway activation between different IBD subtypes (UC vs. CD), still lack large-sample human multi-omics data validation. Whether antioxidant strategies can specifically block eosinophil-mediated pathological damage has not been investigated in stratified clinical studies.

#### 3.7.4 Multidimensional interaction of eosinophils with the mucosal immune cell network

EOS are no longer viewed as simple effector cells but as important regulatory nodes in the mucosal immune network, forming complex interaction loops with both innate and adaptive immune cells.

- Interaction with CD4^+^ T lymphocytes: activated EOS possess antigen-presenting capabilities. They can take up intestinal antigens, express MHC-II and CD80/CD86 co-stimulatory molecules, and directly promote CD4^+^ T cell proliferation. A noteworthy bidirectional effect: EOS can promote Th2 differentiation, but in the IBD microenvironment, they can also regulate the function of Th1 and Th17 cells, enhancing the secretion of IFN-γ and IL-17A, building a bridge between type 2 immunity and classical autoimmune inflammatory pathways (237). This mechanism can explain some clinical phenomena: IBD patients can simultaneously have elevated Th1/Th17 and Th2 cytokines, and these two types of immune responses cannot be simply viewed as antagonistic.
- Positive loop with ILC2s: intestinal epithelial damage releases alarmins that activate ILC2s. ILC2s secrete IL-5 and IL-13, promoting eosinophil survival and activation. Activated EOS further release mediators that stimulate the continuous secretion of IL-33 and TSLP by the epithelium, persistently maintaining ILC2 activation, constituting a stable chronic inflammatory loop (252). Dietary fiber and gut microbiota metabolites can regulate the activity of this loop, suggesting that environmental factors modulate the level of inflammation in IBD through the ILC2-eosinophil axis.
- Regulation of macrophages and dendritic cells: mediators released by EOS remodel macrophage polarization, driving macrophages towards a pro-inflammatory phenotype. Simultaneously, they regulate the maturation of dendritic cells, enhancing antigen-presenting capacity and amplifying the autoimmune response.

Critical reflection: The vast majority of evidence for cellular interactions comes from *in vitro* co-culture systems and mouse models. There are species-specific differences in the surface receptors and transcriptional regulatory networks of human versus mouse intestinal EOS. The number of homeostatic EOS in the mouse intestine is much higher than in humans, so caution is needed when directly extrapolating findings to human IBD, representing a common methodological pitfall in the field.

#### 3.7.5 Eosinophil-driven intestinal fibrosis and stricture formation: an important, underappreciated effector pathway in Crohn’s disease

Fibrotic strictures are the most serious complication of Crohn’s disease, leading to intestinal obstruction and ultimately requiring surgical resection of the affected bowel segment. For a long time, research on intestinal fibrosis has focused on myofibroblasts, TGF-β, and macrophages. The role of EOS in remodelling the extracellular matrix has long been neglected. A 2021 review by the Jacobs team and subsequent clinical specimen studies have confirmed that the number of activated EOS is significantly increased in the deep tissues of the intestinal wall in fibrostenotic Crohn’s disease. EOS can release TGF-β1, platelet-derived growth factor, and connective tissue growth factor, directly stimulating the proliferation and collagen synthesis of resident intestinal fibroblasts (62, 253). Unlike superficial mucosal inflammation, fibrosis-mediating EOS infiltrate the intestinal submucosa and muscularis propria. Conventional superficial biopsies can easily miss this, leading to clinical underestimation of their role.

This finding has significant translational implications: stratified treatment of IBD not only requires controlling superficial mucosal ulcerative inflammation but also intervening in eosinophil infiltration in the deep intestinal wall to block fibrosis progression. The effects of current mainstream biologics (anti-TNF-α, vedolizumab, ustekinumab) on EOS in the deep lamina propria are heterogeneous. Real-world cohort observations have found that responders to ustekinumab treatment experience a decrease in peripheral eosinophil levels, whereas no consistent change is seen in the adalimumab-responsive population, suggesting that different targeted drugs have differential regulatory effects on eosinophil pathways, which could serve as future markers for stratified medication use (254).

#### 3.7.6 Context-dependent inflammatory and tissue-protective functions

IBD illustrates why eosinophil function cannot be inferred from abundance alone. Experimental and tissue-resolved studies show that activated eosinophil states can contribute to host defence and immune regulation, while other states amplify barrier injury, oxidative stress, and fibrosis. Gurtner et al. identified active intestinal eosinophil programs with context-dependent effects in colitis (49). These observations support tissue adaptation and state heterogeneity; they should not be simplified into a universal protective-versus-pathogenic binary.

#### 3.7.7 Overlap, differentiation, and comorbidity mechanisms between IBD and the eosinophilic gastrointestinal disease spectrum

Clinically, it is essential to strictly distinguish between primary eosinophilic colitis (EoC) and IBD with concomitant mucosal eosinophilia. The pathological manifestations of the two overlap, but there are significant differences in pathogenesis and treatment strategies, leading to long-standing diagnostic confusion (255). eosinophilic gastrointestinal diseases (EGIDs) (including eosinophilic esophagitis EoE, eosinophilic gastroenteritis) are primarily driven by food antigen-driven pure type 2 immunity; classic IBD is centered on dysbiosis-driven Th1/Th17 responses. However, epidemiological studies have confirmed a significant comorbidity risk between the two types of diseases: the prevalence of EoE in IBD patients is significantly higher than in the general population, and bidirectional Mendelian randomization analyses suggest shared susceptibility genes and immune pathways (256, 257).

The shared pathways between the two mainly include epithelial barrier defects (the epithelial barrier hypothesis) and the IL-33/ILC2/eosinophil axis. The core differences lie in significant gut dysbiosis and autoreactive T cell activation in IBD, while EGIDs lack persistent Th17 activation. A key clinical differentiating marker is the distribution pattern of MBP: paediatric population studies suggest that MBP staining patterns help distinguish simple EoE from secondary esophageal eosinophil infiltration in IBD (258). At the same time, caution is warranted: some early, atypical IBD cases initially only present with mucosal eosinophilia and may be misdiagnosed as primary eosinophilic enteritis. As the disease progresses, they may gradually evolve into typical CD/UC, suggesting that eosinophilia can serve as a prodromal phenotype for some IBD cases (259).

#### 3.7.8 Clinical translation controversies, biomarker value, and targeted therapy prospects

##### 3.7.8.1 Eosinophils as prognostic and predictive biomarkers for treatment efficacy

Multiple retrospective cohorts have confirmed that elevated peripheral blood EOS at initial diagnosis are associated with a more severe disease course and higher risk of complications in IBD. Tissue mucosal eosinophil density can predict the success rate of induction of remission with vedolizumab and mirikizumab (260, 261). However, there are still many negative results. Sources of heterogeneity include the biopsy site (superficial biopsy vs. full-thickness specimen), the disease phase at the time of biopsy, and differences between children and adults. The prognostic value of mucosal eosinophil infiltration in paediatric IBD is not yet consistent, suggesting that age-related differences in immune development modulate eosinophil effects (262). Fecal EDN is an extremely promising non-invasive marker, superior to a simple peripheral blood count, as it can reflect the degranulation level of locally activated EOS in the gut. Future prospective multicentre cohorts are needed to establish cut-off values and incorporate this marker into clinical monitoring systems.

##### 3.7.8.2 Opportunities and controversies of targeted eosinophil therapy

Monoclonal antibodies against IL-5, IL-5Rα, and Siglec-8 have been widely used in asthma and EGIDs, but consistent positive results have not yet been achieved in IBD clinical trials. Potential reasons include three points:

1. EOS in IBD have dual functions, and comprehensive depletion may do more harm than good;
2. Only a subset of IBD patients have significant eosinophil-driven inflammation, belonging to a specific inflammatory endotype. Clinical trials that do not stratify patients will inevitably yield overall negative endpoints;
3. EOS infiltrating the deep intestinal wall are more difficult to target with antibody drugs compared with circulating EOS.

From this, a key clinical perspective emerges: IBD should not be viewed as a single disease but should be divided into molecular endotypes based on the characteristics of immune cell infiltration. The subgroup with high eosinophil infiltration is suitable for anti-type 2 immune and anti-eosinophil targeted therapy. The future direction is no longer universal eosinophil clearance but specific inhibition of the activated eosinophil ROS pathway and degranulation process, preserving the function of homeostatic protective EOS.

#### 3.7.9 Evidence limitations and translational priorities in IBD

The major challenge in IBD is to distinguish eosinophil states that mark active inflammation from those involved in homeostasis or repair. Blood counts correlate imperfectly with mucosal biology, deep-wall disease is difficult to sample, and animal colitis models vary substantially. For these reasons, broad eosinophil depletion cannot be assumed to benefit unselected IBD. Future studies should combine spatial phenotyping, longitudinal biopsies, and treatment-response data to define clinically actionable endotypes.

### 3.8 Participation of eosinophils in other related autoimmune diseases

The connective tissue disease spectrum centered on a fibrotic phenotype has long been regarded as a Th1-dominant, vascular injury-driven autoimmune disease model. However, over the past two decades, evidence from clinical cohorts, histopathology, and multi-omics has continuously challenged this classic paradigm, suggesting that EOS are not confined to allergic diseases and EGPA but are widely involved in tissue remodelling, chronic inflammation, and immune homeostatic imbalance in various heterogeneous autoimmune diseases such as sclerosing connective tissue diseases and some exocrine gland autoimmune diseases. This section focuses on three diseases—systemic sclerosis, eosinophilic fasciitis, and localized scleroderma (morphea)—that have significant clinical overlap and share pathological fibrotic features. It critically examines the dual role of EOS in disease occurrence and development, existing controversies, unresolved core scientific questions, and compares the similarities and differences in eosinophil activation patterns among these diseases. The discussion also briefly extends to eosinophil-related evidence in other autoimmune diseases such as primary Sjögren’s syndrome to establish a unified pathological framework.

#### 3.8.1 Systemic sclerosis

Systemic sclerosis (SSc) is a disabling autoimmune connective tissue disease characterized by the triad of autoantibody production, microvascular endothelial injury, and progressive tissue fibrosis. Based on the extent of skin involvement, it is divided into limited cutaneous systemic sclerosis and diffuse cutaneous systemic sclerosis, with interstitial lung disease (SSc-ILD) being the leading cause of death. For a long time, SSc was defined as a typical Th1/Th17-skewed disease. However, accumulating evidence confirms that local and circulating eosinophil activation is a hallmark immune feature of a significant subset of SSc and is closely associated with adverse outcomes such as skin ulcers and interstitial lung disease, constituting a highly controversial scientific proposition in the current field.

Early studies already observed that serum levels of eosinophil cationic protein (ECP) in SSc patients were four times higher than those of healthy controls, directly supporting the interpretation that EOS are in a state of persistent activation in SSc, rather than simply increased in number (263). It is worth emphasizing that an absolute increase in peripheral blood eosinophil count is not universal in SSc; fewer than 5% of patients exhibit persistent peripheral eosinophilia, suggesting that *in-situ* tissue infiltration and degranulation are more critical biological markers than circulating eosinophilia (264). Skin biopsy results show that infiltrating EOS can be detected in the dermis of active sclerotic skin lesions. These cells release granule proteins such as MBP, ECP, and eosinophil-derived neurotoxin (EDN), which directly induce fibroblast proliferation and the massive synthesis of type I and type III collagen. Granule toxins simultaneously damage the microvascular endothelium, exacerbating the characteristic vasculopathy of SSc, forming a vicious cycle of “endothelial injury–inflammatory cell recruitment–fibrosis amplification” (265, 266).

The lungs are the most important target organ for eosinophil-mediated injury. Elevated EOS in the bronchoalveolar lavage fluid (BALF) of SSc-ILD patients is an independent adverse prognostic factor. Individuals with BAL eosinophilia experience a significantly faster rate of lung function decline and a higher risk of long-term respiratory failure (267, 268). A key, unresolved core contradiction lies in the fact that SSc-ILD has two distinct subtypes of alveolar inflammation: neutrophil-predominant and eosinophil-predominant, which respond differently to existing antifibrotic drugs. Animal experiments have found that in the Fra-2 transgenic SSc model, pirfenidone treatment paradoxically upregulates lung IL-4 levels and promotes the recruitment of EOS into lung tissue, suggesting that existing antifibrotic drugs cannot block the type 2 inflammatory pathway and have limited efficacy in the eosinophilic inflammation-dominant SSc-ILD subgroup (269). This phenomenon strongly suggests inherent disease heterogeneity within SSc. Molecular subtype stratification based on eosinophil activation status is possible and represents an important entry point for future precision therapy.

At the molecular mechanism level, the epithelial barrier injury hypothesis provides a unified theoretical framework for eosinophil involvement in SSc. Disruption of the body’s skin, airway, and digestive tract epithelial homeostasis leads to the release of the alarmin IL-33. Through the ST2 receptor pathway, IL-33 simultaneously drives Th2 cell differentiation and eosinophil survival, chemotaxis, and degranulation (119, 122, 270). There is a significant overlap in gene expression profiles between esophageal disease in SSc and eosinophilic esophagitis (EoE), corroborating that epithelial barrier breakdown acts as an upstream initiating event, activating tissue type 2 inflammation, inducing eosinophil infiltration, and thereby promoting smooth muscle fibrosis and dysmotility in the digestive tract. This explains the pathological basis for the high prevalence of esophageal dysfunction in SSc (271, 272). The discovery of IgE-type autoantibodies further completes the regulatory network: some SSc patients have anti-nuclear antigen IgE autoantibodies, which can cross-link FcϵRI on the eosinophil surface, initiating autoimmune degranulation independent of exogenous allergens. This provides breakthrough evidence for “self-antigen-driven eosinophil activation,” breaking the traditional belief that EOS only respond to exogenous allergens (273).

Despite the above evidence gradually building a pathway model for eosinophil involvement in SSc pathogenesis, several key critical controversies still exist in the field:

1. The causality dilemma: existing cross-sectional studies struggle to distinguish whether eosinophil activation is a trigger for the initiation of fibrosis or a secondary inflammatory response following chronic tissue injury. Some scholars propose that the persistent fibrotic process releases matrix degradation fragments and damage-associated molecular patterns (DAMPs), passively recruiting EOS, and that eosinophil infiltration is merely a bystander inflammatory cell, not an initiating factor for fibrosis.
2. The duality of cell function: EOS simultaneously secrete galectin-10 (Galectin-10), which has the potential to inhibit excessive T-cell activation and maintain immune tolerance. Serum galectin-10 levels are decreased in SSc patients, suggesting that EOS, under different microenvironments, possess dual phenotypes of promoting fibrosis and immune regulation, with the ultimate effect depending on the local cytokine microenvironment (274).
3. The clinical translation dilemma: clinical trials of monoclonal antibodies targeting IL-5/IL-5R have not yet achieved consistently positive results in SSc, suggesting that simply depleting EOS is insufficient to block the complex, multi-pathway process. EOS are an important node in the fibrotic network but not the sole driving link.

#### 3.8.2 Eosinophilic fasciitis (Shulman disease)

Eosinophilic fasciitis was first reported by Shulman in 1974. It is a rare sclerosing autoimmune connective tissue disease easily confused with SSc. The core clinical features are symmetrical, painful swelling of the extremities, progressing to wooden sclerosis of the subcutaneous tissue and fascia. Typical laboratory findings include peripheral blood eosinophilia and hypergammaglobulinemia. Key differentiating points from SSc include the usual absence of Raynaud’s phenomenon, sclerodactyly, nail fold capillary abnormalities, and a significantly lower incidence of visceral involvement (275–277).

The histopathological features intuitively highlight the central role of EOS: the characteristic lesion of EF is located in the deep fascia, where the fascial layer exhibits diffuse infiltration of a large number of lymphocytes, plasma cells, and EOS, accompanied by fascial collagen hyperplasia and thickening. The lesion is less confined to the superficial dermis. This pathological distribution pattern allows for direct differentiation from SSc and morphea (278, 279). The serum of patients with active disease possesses strong EOS chemotactic activity. *In vitro* experiments have confirmed that patient serum can efficiently induce directed eosinophil migration, suggesting that a gradient of circulating chemotactic factors continuously mediates the recruitment of peripheral EOS to fascial lesions (280). The current mainstream hypothesis is that external triggers such as trauma, strenuous exercise, or environmental exposure induce fascial tissue injury, releasing alarmins that activate local innate immunity. This drives the secretion of eosinophil chemokines such as CCL11 and CCL24, recruiting circulating EOS to infiltrate the fascia. Infiltrated EOS undergo massive degranulation, releasing granule toxins that induce sustained activation of fascial fibroblasts and excessive extracellular matrix deposition. This also promotes increased vascular permeability, forming the characteristic limb oedema seen in the early disease stage. As the oedema persists and progresses, it gradually transforms into irreversible fibrosis.

The differentiation between EF and SSc has always been a clinical and pathological challenge. Both share the phenotypes of skin sclerosis and fibrosis, but the dynamics of EOS are fundamentally different. Peripheral blood eosinophilia in EF mostly appears in the early active phase of the disease and quickly declines after glucocorticoid treatment. In contrast, SSc rarely presents with persistent peripheral eosinophilia, with *in-situ* tissue activation being the predominant feature (264). From a disease spectrum perspective, an increasing number of case reports describe overlap syndromes between EF and morphea. Some patients, upon long-term follow-up, can transform into classic localized scleroderma. This suggests that EF, morphea, and SSc together constitute a continuous disease spectrum of sclerosing connective tissue diseases. The intensity of eosinophil activation determines the phenotypic bias: high eosinophil activation tends to manifest as EF; mild, localized eosinophil infiltration is more likely to develop into morphea; and cases with only localized, low-level eosinophil activation and predominantly vascular injury manifest as classic SSc (281, 282).

Current EF research faces significant shortcomings that constrain mechanistic elucidation. First, the extremely low incidence of the disease results in a lack of large-scale prospective cohorts. The vast majority of evidence comes from single-center case series, lacking long-term follow-up immune dynamic monitoring data. Second, there is no standardized animal model that mimics human EF fascial lesions, making it impossible to conduct cell-specific knockout experiments to clarify the causal role of EOS. Finally, the autoantigen in EF has not been identified to date. It remains impossible to answer whether eosinophil activation is an adaptive immune response driven by autoantigens or simply an innate immune response triggered by tissue injury. At the clinical level, glucocorticoids remain the first-line treatment. Some refractory cases treated with IL-5 targeted therapy have achieved case-level remission, but there is a lack of randomized controlled study support. There is an urgent need to explore the value of eosinophil-targeted therapies relying on multicentre registry studies (275, 283).

#### 3.8.3 Localized scleroderma (morphea)

Localized scleroderma is characterized by localized dermal fibrosis and sclerosis of the skin, without systemic visceral involvement. Subtypes include plaque, linear, generalized, and pan-sclerotic morphea. Previous research on morphea centered on T-cell-mediated autoimmune injury. However, recent histopathological studies have confirmed that varying numbers of infiltrating EOS are commonly present in active morphea skin lesions. The degree of eosinophil infiltration in generalized and pan-sclerotic morphea lesions is significantly higher than in localized plaque subtypes, suggesting that EOS participate in lesion expansion and fibrosis progression (284, 285).

From a pathological spectrum perspective, morphea lies in an intermediate position between EF and SSc. Compared with EF, eosinophil infiltration in morphea is confined to the dermis, rarely involving the deep fascia, and peripheral blood EOS are mostly normal. Compared with SSc, morphea lacks significant microvascular disease, and the fibrosis is predominantly localized to skin lesions. Overlap cases further corroborate the intrinsic link between the three. Clinically observable cases of morphea and EF coexistence or mutual transformation suggest that the three share a basic pathway of “tissue injury–type 2 inflammatory activation–eosinophil recruitment–matrix remodelling.” The depth of the target tissue, the intensity of immune activation, and the degree of vascular involvement determine the final clinical phenotype (286). Mechanistically, activated EOS within the lesion release cytotoxins, stimulate dermal fibroblasts, and inhibit the expression of matrix metalloproteinases, disrupting the balance of collagen synthesis and degradation. Simultaneously, EOS secrete IL-13, further amplifying local type 2 inflammation and forming a positive feedback loop that maintains chronic fibrosis.

It is worth critically considering that eosinophil infiltration in morphea lesions is highly heterogeneous. Approximately 30% of active lesions have almost no EOS, suggesting that morphea also has molecular subtypes: one dominated by eosinophil-mediated type 2 inflammation, and another driven by the classical Th1/IFN-γ pathway. These two subtypes potentially have different treatment responses. Currently, clinical treatment is not stratified based on immune cell characteristics. Future research needs to utilize spatial transcriptomic technologies to analyze the interaction network between EOS and other immune cells and stromal cells within the lesion, distinguishing therapeutic targets for different subtypes.

#### 3.8.4 Dermatomyositis/polymyositis, eosinophilia–myalgia syndrome, and other connective-tissue disorders

EOS are not a defining pathological feature of idiopathic dermatomyositis or polymyositis, and current evidence is insufficient to assign them a major disease-driving role. Occasional eosinophil-rich myositis should prompt careful differential diagnosis, including eosinophilic myositis and systemic eosinophilic disorders. Eosinophilia–myalgia syndrome (EMS) is included here as a mechanistic comparator rather than a prototypic autoimmune disease: it is an exposure-associated inflammatory and fibrosing syndrome in which eosinophilia, fascia/muscle involvement, and immune dysregulation can coexist (287). This distinction prevents the broad category of “eosinophil-associated inflammation” from being conflated with established autoimmunity.

#### 3.8.5 Pediatric autoimmune disease: an evidence gap rather than an adult analogue

Age-specific eosinophil biology is under-studied in childhood-onset SLE, juvenile idiopathic arthritis (JIA), and juvenile dermatomyositis. JIA transcriptomic datasets have identified immune-cell-related signatures (101), but they do not establish an eosinophil-specific causal pathway, and adult rEOS/iEOS markers or ELR cut-offs should not be extrapolated to children. Pediatric studies should account for developmental immunology, age-dependent reference ranges, atopy, infection, and corticosteroid exposure, and should prioritize paired blood–tissue phenotyping where clinically feasible.

Primary Sjögren’s syndrome (pSS) is an autoimmune disease characterized by lymphocytic infiltration of exocrine glands. The classic immune framework is dominated by the type I interferon pathway and B cell abnormalities. However, cohort studies have found that some pSS patients develop complications such as chronic eosinophilic pneumonia, eosinophilic gastroenteritis, and eosinophilic sialodochitis. Patients with such complications exhibit significantly elevated serum eosinophil counts and eosinophil/lymphocyte ratios (288, 289). Local eosinophil infiltration in salivary gland lesions can exacerbate ductal epithelial damage and accelerate acinar fibrosis. Elevated peripheral blood eosinophil-related markers are associated with the risk of pulmonary involvement in pSS. It is noteworthy that EOS rarely appear in the typical basal salivary gland lesions of classic Sjögren’s syndrome, only manifesting when accompanied by specific organ complications. This suggests that EOS are not the basic disease driver in pSS but rather a consequence of secondary tissue injury or the superimposition of comorbid type 2 inflammation (290).

Systemic lupus erythematosus (SLE) is traditionally regarded as a type I interferon-dominant disease. However, subgroup analysis suggests that SLE subgroups with low IFN signature expression have enrichment of eosinophil-related genes. Meanwhile, SLE patients can develop eosinophilic pleural effusion and eosinophilic gastroenteritis, mostly occurring during the active, organ-involvement phase of the disease. Unlike sclerosing diseases, EOS in SLE are more involved in immune complex-mediated local tissue inflammation, and their pro-fibrotic effects are not prominent (290).

#### 3.8.6 Cross-disease synthesis

Across SSc, eosinophilic fasciitis, morphea, myositis-spectrum disorders, and pediatric autoimmune disease, the signal for eosinophil involvement ranges from biologically plausible to clinically prominent, but the quality of evidence is generally lower than in EGPA or eosinophil-rich blistering disease. The common translational need is longitudinal, tissue-resolved evidence that separates eosinophil recruitment secondary to injury from eosinophil programs that are necessary for disease propagation.

## 4 Eosinophil-targeted therapies: mechanism, evidence, and risk–benefit trade-offs

Based on the unique pathogenic mechanisms of EOS in autoimmune diseases clarified in the preceding text—including heterogeneous activation of EOS subsets, degranulation and release of toxic mediators, formation of extracellular traps (EETs), disordered epithelial-immune interactions, and tissue remodelling driving effects—targeted EOS-specific inhibitors have become the core precision intervention method for blocking the autoimmune Th2-type inflammatory cascade and breaking the autoimmune tolerance imbalance. In contrast to the non-specific immunosuppression of traditional broad-spectrum immunosuppressants, the systemic toxic side effects of glucocorticoids, and the immune balance disruption caused by conventional immunosuppressive agents, the new generation of EOS-targeted inhibitors targets the core molecular pathways of EOS development, activation, recruitment, and effector release. This achieves precision blockade of pathogenic EOS inflammation while maximally preserving the physiological functions of EOS innate immune defence, representing a core breakthrough direction for precision therapy of autoimmune inflammation (291, 292). This chapter focuses on the molecular structure, pharmacological mechanisms, target specificity, technical iteration logic, and existing academic controversies of various targeted EOS inhibitors. It does not reiterate the clinical therapeutic efficacy for various autoimmune diseases but concentrates on deeply analyzing the characteristics, limitations, and future research and development trends of the inhibitors from the perspectives of basic pharmacology and translational medicine. This provides theoretical support for the mechanistic research of precision EOS-targeted intervention in autoimmune diseases.

### 4.1 pathway targeted inhibitors: IL-5/IL-5Rα monoclonal antibody inhibitors

IL-5 is the specific core cytokine governing the biological behavior of EOS and is currently the most mature and specific target for EOS-targeted drug development. Unlike broad-spectrum Th2 inflammatory factors such as IL-4 and IL-13, IL-5 specifically regulates only the differentiation and maturation of EOS in the bone marrow, the survival homeostasis of peripheral blood EOS, the activation and enrichment of tissue EOS, and degranulation effects. It is the sole key regulatory molecule for the pathogenic activation of EOS (293, 294). Under physiological conditions, low-level expression of IL-5 maintains the homeostatic distribution and immune defence functions of EOS in the intestinal and respiratory tract mucosae. Under the imbalanced state of autoimmune type 2 inflammation, Th2 cells and type 2 innate lymphoid cells (ILC2s) aberrantly hypersecrete IL-5, driving excessive EOS proliferation, activation, tissue infiltration, and toxic mediator release, mediating autoimmune tissue injury (295, 296). Currently, the mainstream IL-5 pathway-targeted inhibitors in clinical and basic research fall into two categories: soluble IL-5-neutralizing monoclonal antibodies and IL-5 receptor α (IL-5Rα)-targeted depleting monoclonal antibodies. Their mechanisms of action are fundamentally different, forming a differentiated targeted intervention system.

Depemokimab is a long-acting anti-IL-5 antibody designed for prolonged pathway suppression. Its pharmacological effect is sustained IL-5 neutralization; it has not been shown to selectively prevent conversion into a human CD62L−/CD101+ inflammatory eosinophil subtype. The CD62L/CD101 framework derives predominantly from mouse studies and should be used only as a preclinical conceptual model when discussing human drug action. Any subset-selective interpretation of depemokimab therefore remains hypothetical pending human tissue and single-cell validation.

Mepolizumab is the world’s first approved humanized IgG1-type anti-IL-5 monoclonal antibody and a landmark drug for EOS-targeted therapy. Its molecular design achieves high specificity and high affinity binding to human soluble IL-5, precisely blocking the specific binding of IL-5 to IL-5Rα on the EOS surface, thereby blocking the signal initiation of EOS activation from upstream (297, 298). From the perspective of molecular pharmacological mechanisms, mepolizumab does not directly clear already mature EOS in the body. Instead, by blocking IL-5–dependent survival signals, it reduces the bone marrow output efficiency of EOS, shortens the half-life of peripheral EOS, and inhibits the activation and degranulation of tissue EOS, thereby downregulating the EOS-mediated inflammatory cascade (299, 300). The core advantage of this mode of action is the reversible regulation of EOS homeostasis, rather than complete EOS depletion. It maximally preserves the physiological functions of EOS in anti-parasitic infection and mucosal immune surveillance, circumventing the risk of immune deficiency brought about by complete EOS depletion. This is also the mechanism underlying its superior long-term safety compared with potent depleting inhibitors (301).

Reslizumab is also a humanized anti-IL-5 monoclonal antibody. Its molecular affinity and specificity for IL-5 binding are slightly higher than mepolizumab. The pharmacological mechanisms of the two are highly homologous, but there are subtle pharmacokinetic differences. Reslizumab has a higher neutralization efficiency for circulating free IL-5 and can rapidly reduce peripheral blood EOS counts. However, its tissue penetration is weaker than mepolizumab, limiting its inhibitory effect on tissue-resident activated EOS (302, 303). From a critical analysis of mechanistic defects, both traditional anti-IL-5 antibodies share a common technical bottleneck: they only target soluble free IL-5 and cannot block the autocrine activation loop of membrane-bound IL-5 on EOS. Moreover, their inhibitory effect on activated EOS that have already colonized inflamed tissues and are continuously releasing toxic mediators is delayed. This is the mechanism behind poor drug response and post-treatment inflammatory relapse in some patients with autoimmune diseases (304).

The development of the new-generation long-acting inhibitor depemokimab has broken through the pharmacokinetic shortcomings of traditional anti-IL-5 antibodies. Through Fc segment modification technology, the drug half-life is extended, achieving a super-long dosing interval of once every 6 months (305, 306). At the mechanistic level, depemokimab retains the function of highly specific neutralization of soluble IL-5 while optimizing the molecular structure to enhance tissue penetration capability. This allows for more efficient blockade of IL-5 signal enrichment within the local inflammatory microenvironment. The latest mechanistic research confirms that depemokimab can continuously inhibit the differentiation of EOS into the CD62L^-^ inflammatory subtype. This subtype is the core pathogenic EOS subset mediating tissue injury in autoimmune inflammation. Traditional anti-IL-5 antibodies have a time-limited inhibitory effect on this subtype, whereas the long-acting formulation achieves sustained subtype regulation (307). However, there are still gaps in the mechanistic research of this drug: its blocking efficiency against the formation of EETs and the continuous release of granule proteins remains unclear. Furthermore, whether long-term intervention interferes with mucosal EOS homeostasis balance still requires basic experimental validation (305).

#### 4.1.1 IL-5Rα-targeted depleting inhibitors: the unique mechanism and bidirectional controversy of Benralizumab

Benralizumab is currently the only approved humanized monoclonal antibody targeting IL-5Rα on the EOS surface. Its mechanism of action is completely different from that of IL-5-neutralizing inhibitors, representing a potent depletion strategy for EOS-targeted intervention (308, 309). EOS highly express IL-5Rα on their surface (expression level is more than three times that of basophils), which is the core basis for its specific targeting (295). By specifically binding to IL-5Rα on the surface of EOS and basophils, benralizumab competitively blocks IL-5 signal transduction on one hand, and on the other, mediates antibody-dependent cell-mediated cytotoxicity (ADCC) through its Fc segment, recruiting natural killer cells and macrophages to precisely lyse circulating and tissue-infiltrated activated EOS, achieving near-complete EOS depletion (301, 310).

In terms of mechanistic advantages, benralizumab solves the core shortcoming of traditional anti-IL-5 antibodies: without relying on IL-5 signal blockade, it can directly clear already activated and colonized pathogenic EOS. This completely blocks downstream pathogenic effects such as EOS degranulation, EETs release, and inflammatory factor secretion, offering a unique intervention effect for refractory EOS inflammation mediated by the IL-5 autocrine loop (294). Mechanistic studies in Th2-type autoimmune diseases have confirmed that benralizumab can rapidly clear pathogenic EOS infiltrating skin lesions, mucosae, and vessel walls, blocking EOS-mediated epithelial barrier disruption, vascular endothelial injury, and tissue fibrosis. Its anti-inflammatory potency is significantly superior to that of IL-5-neutralizing inhibitors (311, 312). Simultaneously, the drug has a significant glucocorticoid-sparing effect, capable of breaking the vicious cycle of hormone dependence in autoimmune disease treatment by potently depleting EOS inflammation (313, 314).

From a critical perspective, the potent depletion mechanism of benralizumab has non-negligible inherent defects and academic controversies:

1. The drug non-selectively depletes all EOS, including physiological homeostatic EOS. Long-term intervention may disrupt the mucosal immune barrier and increase the risk of pathogen infection, which is also the core of its long-term safety controversy (301);
2. Recent EOS heterogeneity research has confirmed the existence of protective EOS subsets in the body that participate in tissue repair and immune homeostasis regulation. Non-specific depletion by benralizumab will simultaneously clear protective subsets, potentially interfering with the inflammatory repair process and being detrimental to tissue remodelling and repair after autoimmune injury (315, 316);
3. Some clinical mechanistic studies have found that after benralizumab depletes EOS, some patients still exhibit persistent Th2 inflammatory activation, suggesting that EOS are not the only effector cells of Th2 autoimmune inflammation. Simple EOS depletion cannot completely block the autoimmune inflammatory loop, explaining the mechanism of poor drug response in some patients (304).

### 4.2 Indirect pathway inhibitors targeting EOS activation

Beyond the core IL-5 pathway, the recruitment, activation, degranulation, and effector release of EOS depend on the regulation of multiple upstream signaling pathways. Inhibitors targeting these pathways can indirectly block the pathogenic activation of EOS, forming a multi-dimensional EOS-targeted intervention system. Such inhibitors do not act directly on EOS themselves but suppress the pathogenic biological behavior of EOS by modulating the inflammatory microenvironment, chemotactic signals, and mediator release pathways, preserving the basic physiological functions of EOS. They are suitable for mild-to-moderate EOS-related autoimmune inflammation and combined targeted intervention scenarios (60, 317).

#### 4.2.1 CCR3 chemotactic pathway inhibitors

CCR3 is a specific chemokine receptor on the EOS surface, serving as the target mediating the directed recruitment of EOS from peripheral blood to inflamed tissues. Its ligands, Eotaxin-1/2/3, are aberrantly highly expressed in the autoimmune inflammatory microenvironment, driving EOS tissue infiltration and local inflammation amplification (318). CCR3 small molecule antagonists can specifically block the Eotaxin-CCR3 signaling axis, inhibiting the directed migration and tissue colonization of EOS, thereby blocking EOS-mediated local autoimmune injury from a spatial dimension (60). Mechanistic studies confirm that these inhibitors can significantly reduce the density of EOS infiltration in target tissues of autoimmune diseases, decrease the local release of toxic granule proteins such as ECP and MBP, and inhibit epithelial barrier injury and local inflammatory cascades (319).

The core advantages of this class of inhibitors are strong target specificity and extremely low systemic side effects. They only block the EOS chemotactic pathway, without affecting EOS bone marrow development or peripheral homeostasis, achieving a precision regulation of “functional inhibition rather than cell clearance” (317). However, their limitation lies in the singularity of the mechanism of action. They can only block EOS recruitment and cannot inhibit the activation, degranulation, and EETs release of EOS that have already infiltrated the tissue. Their intervention effect on chronic, persistent autoimmune EOS inflammation is limited. They are only suitable for early prevention of inflammation and control of mild inflammation, unable to resolve refractory EOS-driven autoimmune injury (60). Currently, this class of inhibitors is still in the basic research and preclinical research stages, not yet widely applied to autoimmune diseases. The bottleneck lies in insufficient tissue penetration of the drug, making it difficult to effectively block EOS chemotactic signals in deep tissues (317).

#### 4.2.2 OXE receptor pathway inhibitors

5-Oxo-eicosatetraenoic acid (5-oxo-ETE) is the most potent lipid chemotactic activating factor for EOS. By specifically binding to the OXE receptor on the EOS surface, it drives rapid EOS activation, degranulation, and inflammatory factor secretion, representing a key pathway in the lipid metabolism regulation of autoimmune EOS inflammation (320). OXE receptor inhibitors can specifically block the EOS activation signal mediated by 5-oxo-ETE, inhibiting excessive EOS activation induced by lipid metabolic disorders and blocking the positive feedback loop between lipid inflammation and EOS inflammation (320).

Recent mechanistic studies have discovered that significant lipid metabolism abnormalities exist in autoimmune type 2 inflammation. The abnormal accumulation of 5-oxo-ETE is an important non-immune driving mechanism for the sustained activation and persistent inflammation of EOS. Traditional anti-IL-5 inhibitors cannot regulate this lipid pathway, which is also an important reason for treatment resistance in some patients (320). Therefore, OXE receptor inhibitors can serve as complementary targets to traditional IL-5-targeted drugs, filling the gap in intervention for EOS activation mediated by lipid metabolism. However, current research in this field still has significant shortcomings: the OXE receptor is also expressed at low levels in neutrophils and monocytes, leading to slight off-target effects of the inhibitors. Moreover, its regulatory mechanisms on EOS EETs formation and tissue remodelling have not been fully elucidated, limiting its clinical translational application (320).

### 4.3 Novel precision targeting strategies: subset-specific targeting and gene-targeted intervention

With the development of EOS heterogeneity, single-cell sequencing, and spatial transcriptomics technologies, the limitations of traditional “pan-EOS targeting” have been gradually revealed. The new generation of EOS-targeted inhibitor research and development focuses on the precision targeting of pathogenic EOS subsets and precise regulation at the genetic level. This breaks through the non-specific defects of existing drugs, becoming the frontier direction for precision targeted therapy of autoimmune diseases (208, 315).

#### 4.3.1 Pathogenic EOS subset-targeted inhibitors

The latest research has confirmed that there is significant phenotypic and functional heterogeneity among EOS in the human body. CD62L^-^ inflammatory EOS (iEos) are the core pathogenic subset in autoimmune inflammation, specifically enriched in autoimmune-injured tissues, highly expressing toxic granule proteins, inflammatory factors, and chemotactic receptors, driving inflammation amplification and tissue injury. In contrast, CD62L^+^ homeostatic EOS mainly participate in mucosal immune defence and tissue repair, without pathogenicity (208, 307). Traditional IL-5 pathway inhibitors do not distinguish between the two types of EOS, leading to an imbalance between treatment efficacy and safety.

Novel subset-targeted inhibitors are being developed based on iEos-specific surface markers. They can precisely identify and inhibit the pathogenic EOS subset while preserving the physiological functions of homeostatic EOS. Currently, basic research has screened iEos-specific highly expressed membrane molecule targets. Monoclonal antibodies and small molecule inhibitors can be used to specifically block the activation and effector release of iEos, achieving targeted intervention of “precision de-inflammation, preservation of homeostasis” (208). However, bottlenecks still exist in this field: the specificity of EOS subset markers still needs validation. The molecular phenotypes of iEos vary in different autoimmune diseases, and a universal subset-targeted drug has not yet been developed. Furthermore, the pharmacodynamic and pharmacokinetic studies of subset targeting are still in their infancy (315).

#### 4.3.2 Gene and antisense nucleic acid targeting technologies

Gene therapy and antisense oligonucleotide (ASO) technology provide entirely new dimensions for EOS-targeted intervention, enabling the regulation of the expression of key pathogenic genes in EOS at the genetic level, achieving long-acting, precise inflammatory control (209). Existing research focuses on targeting EOS-specific pathogenic genes (such as those encoding ECP, MBP, IL-5Rα). Through gene silencing and transcriptional regulation, the synthesis of EOS toxic mediators and the activation signal transduction can be inhibited, blocking EOS pathogenicity at the root (209).

The core advantages of this technology are long-acting efficacy and specificity. It can achieve long-term suppression of the pathogenic functions of EOS with a single intervention, circumventing the drawbacks of repeated dosing of antibody drugs. Moreover, it can precisely target EOS-specific genes without systemic immunosuppressive side effects (209). However, its translational bottlenecks are significant: the tissue targeting of gene delivery systems is insufficient, making it difficult to precisely act on EOS residing in inflamed tissues. The inflammatory microenvironment of autoimmune diseases affects gene transfection efficiency. Furthermore, the immunological safety and off-target effects of long-term gene regulation still lack systematic validation, and the technology is currently limited to animal experiments and basic research stages (209).

### 4.4 Limitations of current eosinophil-targeted strategies

Three limitations recur across agents. First, peripheral eosinophil depletion does not guarantee suppression of tissue-resident or IL-5-independent programs. Second, deeper depletion is not synonymous with greater clinical benefit; MANDARA supports comparable remission efficacy between benralizumab and mepolizumab despite different depletion depth (24). Third, long-term effects on mucosal defence, tissue repair, infection susceptibility, and malignancy risk remain incompletely characterized in autoimmune populations. Available trials have not established a specific malignancy signal, but absence of a demonstrated signal is not equivalent to proof of lifelong safety.

Cost-effectiveness and patient selection are also underdeveloped outside established indications. A precision strategy should require evidence that EOS are biologically active in the target disease and clinically relevant to the patient’s phenotype. Candidate selection variables include eosinophil count trajectory, tissue infiltration or degranulation markers, comorbid eosinophilic disease, organ involvement, glucocorticoid burden, and prior treatment response. These principles are summarized in **Table 4**. Findings from eosinophilic airway diseases, including bronchiectasis and COPD, provide comparative context but should not be extrapolated directly to autoimmune disease (321, 322).

## 5 Discussion: what the current evidence supports

The literature supports a narrower and more clinically useful conclusion than a universal “eosinophil-driven autoimmunity” model. EOS can act as important effectors in specific diseases and tissues, most convincingly in EGPA and selected eosinophil-rich dermatoses. In other conditions, their contribution is conditional, secondary, or still uncertain. The unifying principle is context dependence: tissue recruitment, local survival signals, activation state, disease phase, and interactions with epithelial, stromal, and adaptive immune compartments determine whether EOS amplify injury, contribute to fibrosis, or participate in homeostasis and repair.

### 5.1 Causality, therapeutic paradoxes, and evidence calibration

Most mechanistic claims in autoimmune disease remain associative or preclinical. Human genetic analyses such as RA Mendelian randomization can strengthen causal inference at the exposure-trait level but do not identify a cell-level mechanism (20). Conversely, randomized trials in EGPA provide direct evidence that IL-5-axis intervention changes clinically meaningful outcomes (23, 24), although they do not establish which eosinophil state is necessary for response. Throughout the review, we therefore distinguish “associated with,” “supports,” and “demonstrates” according to study design.

The dupilumab paradox illustrates the need for this calibration. Transient blood eosinophilia is reproducible, but a specific compensatory IL-5 transcriptional program has not been demonstrated as the single human mechanism. Reduced tissue trafficking, persistent eosinophilopoiesis, redistribution, and unmasking of latent eosinophilic asthma or EGPA are non-exclusive alternatives. The appropriate clinical implication is monitoring and phenotype assessment, not the assumption that IL-4Rα blockade creates *de novo* eosinophilic autoimmunity.

### 5.2 Fundamental limitations of the evidence base

Three constraints directly limit the validity and generalizability of current conclusions: predominance of correlational evidence, imperfect translation from animal models, and restricted access to representative human tissue. These are not merely future research opportunities; they are present limitations that should temper mechanistic and therapeutic inference.

#### 5.2.1 Temporal causality and human tissue sampling

Cross-sectional blood counts and biopsies rarely establish whether eosinophil activation precedes autoimmune injury or follows tissue damage. Biopsy studies are additionally affected by indication bias, anatomical sampling, treatment exposure, and the difficulty of capturing dynamic lesions. Longitudinal paired blood–tissue sampling, ideally before and after defined therapeutic perturbations, is required to establish temporal sequence and disease-stage dependence.

#### 5.2.2 Subset taxonomy and species translation

Human and mouse EOS share broad biology but differ in receptor repertoire, tissue distribution, and granule components. A particularly important difference is Charcot–Leyden crystal protein/galectin-10, which is abundant in human EOS but absent from mouse EOS. Likewise, human Siglec-8 and murine Siglec-F are functional analogues rather than interchangeable markers (81, 82). Widely cited CD62L/CD101 resident-versus-inflammatory schemes are largely derived from mouse lung studies. Consequently, murine subset labels, EETosis pathways, and IL-33/ST2 responses should be explicitly annotated as preclinical unless independently validated in human tissue.

#### 5.2.3 Model and assay limitations

Current models incompletely reproduce human eosinophil biology. Standard mouse systems cannot model CLC/galectin-10 biology, and baseline eosinophil tissue distribution differs across species. Human studies are constrained by short eosinophil lifespan ex vivo, variable activation during isolation, and limited validated antibody panels for state-specific sorting. These limitations are especially consequential for claims about EETosis, tissue-resident subsets, and drug-selective targeting. Humanized models, organoids, standardized functional assays, and spatially resolved primary-tissue studies are needed to bridge this gap.

#### 5.2.4 Therapeutic risk–benefit and long-term depletion

Long-term eosinophil suppression requires a disease-specific risk–benefit assessment. Existing biologics have generally acceptable safety profiles in approved indications, but autoimmune populations may differ in age, immunosuppressive co-therapy, infection risk, and organ damage. The consequences of lifelong or near-complete eosinophil depletion for mucosal defence, tissue repair, infection, and malignancy remain incompletely defined. **Table 4** therefore distinguishes demonstrated adverse events from theoretical or unresolved long-term risks and emphasizes patient selection rather than indiscriminate depletion.

### 5.3 Priorities for mechanistic and clinical translation

The highest-value next steps are as follows: (i) longitudinal human tissue studies that establish temporal sequence and functional necessity; (ii) a reproducible human eosinophil state taxonomy integrating surface phenotype, transcriptome, chromatin, metabolism, and spatial niche; (iii) trials enriched for biologically defined eosinophil-active endotypes; and (iv) pharmacodynamic monitoring that separates blood depletion from tissue effects. Extracellular vesicles, Siglec-8 biology, immunometabolic states, and durable memory-like programs are promising areas, but each should be advanced with evidence proportional to the maturity of the field rather than presented as established autoimmune mechanisms.

## 6 Conclusion

EOS occupy an immunological interface between type 2 inflammation, tissue injury, adaptive immunity, and repair. Their importance in autoimmune disease is real but uneven: dominant in a limited number of eosinophil-rich diseases, modulatory in others, and uncertain in several commonly discussed settings. The field will progress by replacing pan-eosinophil narratives with tissue- and state-resolved evidence, explicitly separating mouse from human biology and association from causation, and matching therapeutic intensity to a validated eosinophil-active phenotype.
